# The GPCR NMUR-1 mediates neural regulation of energy homeostasis in response to pathogen infection

**DOI:** 10.1128/iai.00313-25

**Published:** 2025-11-03

**Authors:** Phillip Wibisono, Yiyong Liu, Kenneth P. Roberts, Dodge Baluya, Jingru Sun

**Affiliations:** 1 Department of Translational Medicine and Physiology, Elson S. Floyd College of Medicine, Washington State University 53424, Spokane, Washington, USA; 2 Genomics Core, Washington State University 53424, Spokane, Washington, USA; 3 Tissue Imaging, Metabolomics and Proteomics Laboratory, Washington State University 6760, Pullman, Washington, USA; University of California San Diego School of Medicine, La Jolla, California, USA

**Keywords:** G protein-coupled receptor, specificity of innate immunity, neuroimmune regulation, NMU, NMUR-1, energy homeostasis, F_1_F_O_ATP synthase, ATP biosynthesis, *C. elegans*, pathogen infection

## Abstract

A key challenge in immunology is understanding how the innate immune system achieves specificity against diverse pathogens. Our previous work in *Caenorhabditis elegans* identified NMUR-1, a neuronal G protein-coupled receptor homologous to mammalian neuromedin U receptors, as a regulator of pathogen-specific innate immune responses. Here, we used quantitative proteomics and functional analyses to show that NMUR-1 modulates the expression of mitochondrial F_1_F_O_ ATP synthase subunits and regulates ATP levels during infection, linking neuronal signaling to host energy metabolism. Loss of NMUR-1 leads to reduced ATP and reactive oxygen species (ROS) concentrations in infected animals, altering survival outcomes in a pathogen-specific manner. We further demonstrate that ATP availability and its contribution to host defense are neurally controlled by the NMUR-1 ligand CAPA-1 and its source neurons, ASG. These findings uncover a neuroimmune mechanism whereby NMUR-1 regulates energy homeostasis as a determinant of innate immune specificity. Our study also provides mechanistic insights into the emerging roles of conserved NMU signaling in neuroimmune regulation across animal phyla.

## INTRODUCTION

The nematode *Caenorhabditis elegans* is a powerful model for investigating host-pathogen interactions at the whole-animal level. This genetically tractable organism is susceptible to infection from a variety of bacterial and viral pathogens, including some known human pathogens (1–3). Unlike vertebrates and some invertebrates, *C. elegans* lacks an adaptive immune system and professional immune cells, relying instead on innate immune responses and behavioral defenses to combat infection (4, 5). Upon exposure to pathogens, *C. elegans* activates evolutionarily conserved immune signaling pathways, including the mitogen-activated protein kinase (MAPK) pathway, the insulin/IGF-1-like DAF-2 pathway, and the transforming growth factor β (TGF-β) pathway (6–8). Activation of these pathways promotes the expression of diverse defense genes that enable the organism to fight infection (4, 9, 10). However, such responses must be precisely regulated, as inappropriate or excessive immune activation can impair host fitness or survival.

Neuronal G protein-coupled receptors (GPCRs) play a key role in tuning immune responses. For example, animals lacking the neuronal GPCR NPR-1 exhibit increased susceptibility to *Pseudomonas aeruginosa* due to dysregulated gene expression in the PMK-1/p38 MAPK pathway (11). In contrast, loss-of-function mutations in the GPCR OCTR-1 enhance resistance to the same pathogen by upregulating stress response genes in the unfolded protein response (UPR) pathway (12–15). These examples illustrate how distinct neuronal signals can either dampen or promote immune activation depending on the context. We previously identified NMUR-1, a neuronal GPCR homologous to mammalian neuromedin U receptors, as a key regulator of pathogen-specific immune responses in *C. elegans* (16). Loss of NMUR-1 enhances survival against the gram-negative bacterium *Salmonella enterica* by upregulating UPR-related genes but reduces survival against the gram-positive bacterium *Enterococcus faecalis*, owing to diminished expression of C-type lectin genes. These earlier findings revealed a transcriptional mechanism by which NMUR-1 modulates innate immune specificity, but its influence at the proteome level remained unclear.

In this study, we examined how NMUR-1 regulates protein expression and host physiology during infection with *S. enterica* and *E. faecalis*. Comparative proteomic analysis revealed that NMUR-1 deficiency leads to reduced abundance of proteins involved in transmembrane ion transport, particularly subunits of the mitochondrial F_1_F_O_ ATP synthase complex. This multi-subunit enzyme plays a critical role in oxidative phosphorylation, converting the proton gradient generated by the electron transport chain into ATP (17, 18). Indeed, both ATP and reactive oxygen species (ROS) concentrations were significantly reduced in *nmur-1* mutant animals, suggesting that NMUR-1 modulates energy homeostasis as part of the immune response. Genetic or chemical inhibition of F_1_F_O_ ATP synthase in wild-type (WT) animals mimicked the distinct survival phenotypes of *nmur-1* mutants during infection with either pathogen, implicating ATP production as a key downstream effector of NMUR-1-mediated immune specificity. Moreover, we found that both the NMUR-1 endogenous ligand CAPA-1 and its expressing neurons ASG are required to maintain ATP and ROS levels and promote host survival during *E. faecalis* infection. These findings establish the CAPA-1/NMUR-1/ASG signaling axis as a neural regulator of energy metabolism in the context of host defense. Collectively, our data identify NMUR-1-mediated control of ATP levels as a central mechanism by which *C. elegans* coordinates pathogen-specific immune responses.

## RESULTS

### Proteomic profiling reveals ATP synthesis pathway alterations during pathogen infection

**Fig 1:**
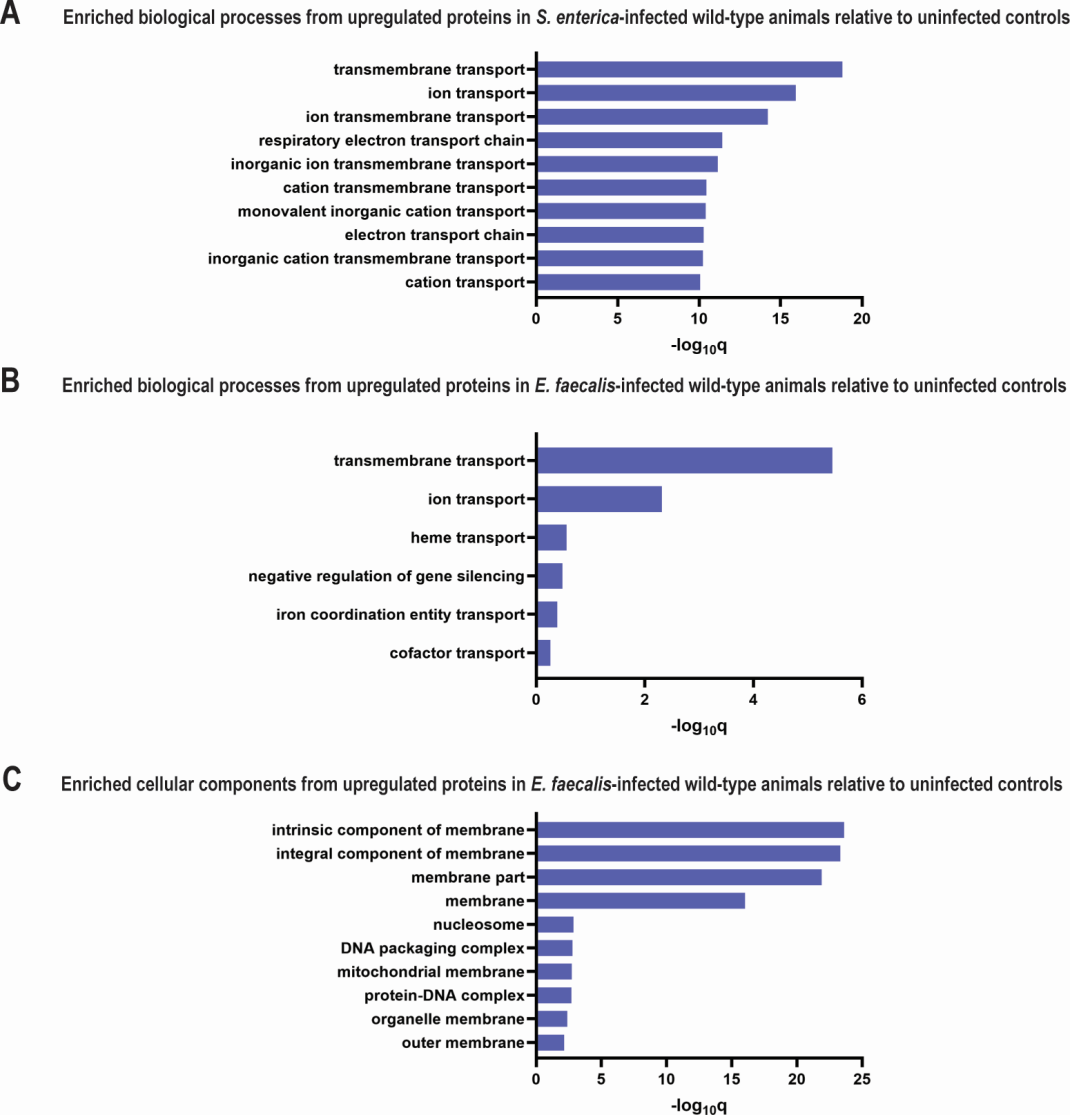
Infection changed the proteomes of WT animals. GO analyses were performed on upregulated proteins in WT animals infected with *S. enterica* (**A**) or *E. faecalis* (**B-C**) relative to uninfected control animals. The graphs show the top 10 most significantly enriched biological processes or cellular components in the infected samples. Bars represent the significance levels of enrichment expressed as -log_10_q values.

To understand how *C. elegans* responds to *S. enterica* and *E. faecalis* infections at the protein level, we compared the proteomes of infected and uninfected WT animals using a label-free quantitative proteomics approach. Five biological replicates of each experimental condition were analyzed with high-resolution nano-HPLC tandem mass spectrometry. Quantifying the proteomes of WT animals exposed to *S. enterica* relative to control animals exposed to *Escherichia coli* (worm food) yielded 2,959 proteins, of which 2,768 were identified with high confidence (1% false discovery rate, FDR) (the Proteomics IDEntifications database [PRIDE] accession number PXD045826). Among these proteins, 373 and 153 were significantly upregulated and downregulated by at least 1.5-fold, respectively. Gene Ontology (GO) analysis of the upregulated proteins revealed 64 significantly enriched biological processes. The top 10 are shown in Fig. 1A, all of which are involved in the nematode’s energy production/electron transport chain. The most significantly upregulated group was “transmembrane transport” (q-value = 1.16E-19), with a notable subgroup being “ATP synthesis-coupled proton transport” (q-value = 1.7E-8). These results indicate that infection with *S. enterica* significantly impacts pathways related to transmembrane transport and ATP synthesis in *C. elegans*. Indeed, *S. enterica* has been reported to cause an increase in reactive oxygen species (ROS) in the intestine of *C. elegans* as part of its pathogenesis (19), and a major source of ROS production in non-photosynthetic cells is as a by-product of ATP production in the mitochondria and as a product of oxidation of various biological molecules such as NADPH (20–22). Therefore, the increase in “ATP synthesis-coupled proton transport” seen in our GO analysis is consistent with an increase in ATP production and induction of ROS during *S. enterica* infection. We also performed GO analysis on the downregulated proteins and did not identify any significantly enriched processes.

We next compared the proteomes of WT animals exposed to *E. faecalis* against control animals exposed to *E. coli* and identified 2,783 proteins with high confidence (1% FDR). Among these proteins, 240 and 120 were significantly upregulated and downregulated by at least 1.5-fold, respectively. GO analysis of the upregulated proteins identified six significantly enriched biological processes (Fig. 1B). Similar to *S. enterica*, *E. faecalis* infection increased the abundance of both “Transmembrane transport” (q-value = 3.54E-6) and “ion transport” (q-value = 4.93E-3) proteins. Further GO analysis of the proteins to identify their relevant cellular components revealed 24 enriched components, the two most significantly upregulated groups were “integral component of membrane” and “intrinsic components of membrane” (q-values = 4.65E-24 and 2.32E-24, respectively) (Fig. 1C). This increase in “integral” and “intrinsic” cell membrane components is consistent with a previous report of epithelial junction integrity being required for the defense against *E. faecalis* (23). GO analysis of the downregulated proteins during *E. faecalis* infection did not yield any significantly enriched biological processes.

Overall, our GO analysis showed that *S. enterica* and *E. faecalis* infections impact pathways related to ATP biosynthesis and energy metabolism in *C. elegans*, indicating that increased energy production is a hallmark of host response to pathogen infection.

### NMUR-1 regulates the levels of ATP synthesis proteins

**Fig 2:**
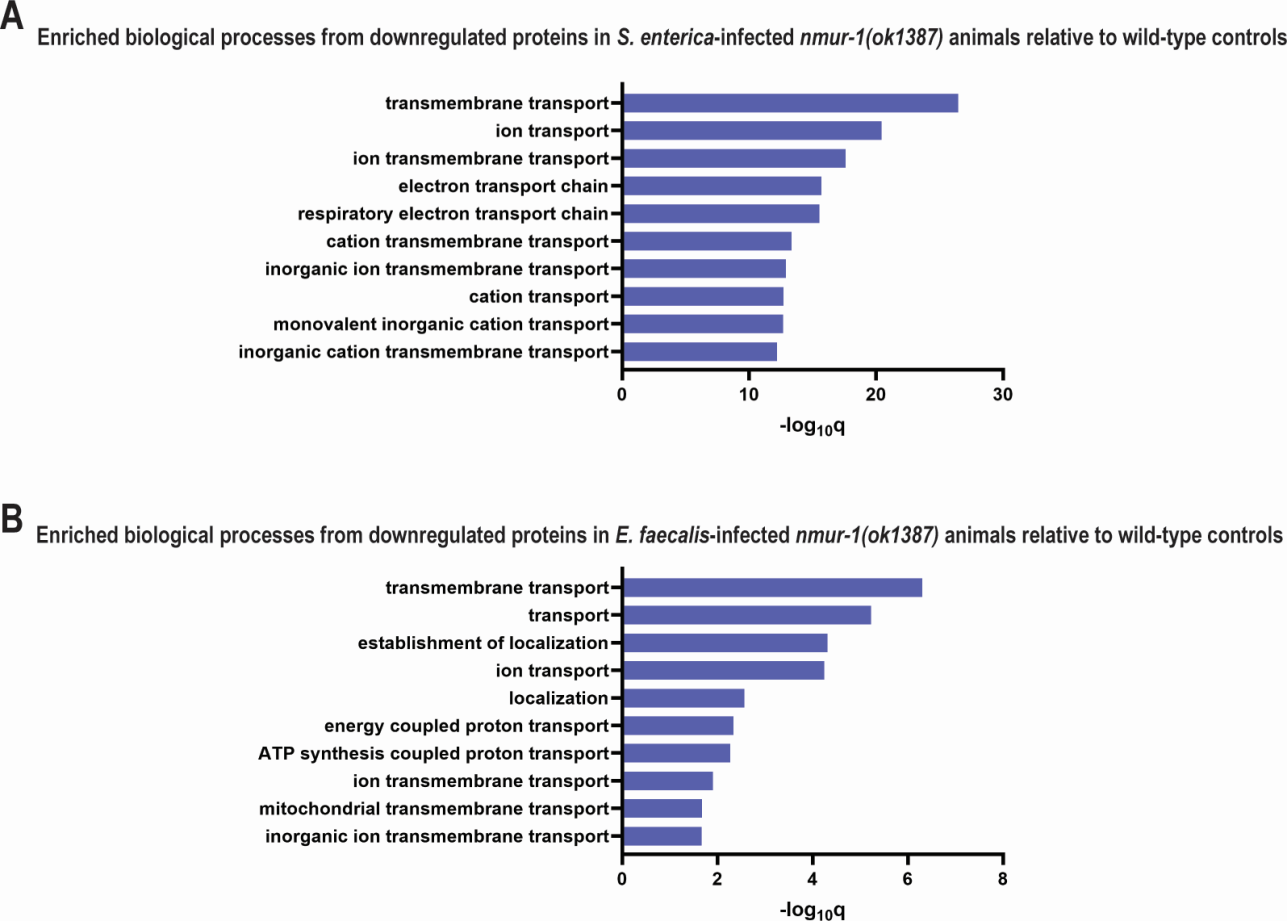
*nmur-1(ok1387*) mutant animals had significant downregulation in ion transport and energy production proteins during infection. GO analyses were performed on downregulated proteins in *nmur-1(ok1387*) animals infected with *S. enterica* (**A**) or *E. faecalis* (**B**) relative to similarly infected WT control animals. The graphs show the top 10 most significantly enriched biological processes in mutant animals. Bars represent the significance levels of enrichment expressed as −log_10_q values.

We next assessed the impact of NMUR-1 loss by comparing proteomes of *nmur-1(ok1387*) mutants and WT animals during pathogen infection. During *S. enterica* infection, 74 proteins were upregulated, and 424 proteins were downregulated in *nmur-1(ok1387*) animals relative to WT animals. GO enrichment analysis on the 74 upregulated proteins found a single enriched biological process, “pseudopodium” (q-value = 1.13E-3); the biological significance is unclear. A similar analysis of the 424 downregulated proteins detected 87 enriched biological processes, the most significant of which was “Transmembrane transport” (q-value = 3.46E-27) (Fig. 2A). This downregulation of transmembrane transport proteins is interesting, as it is juxtaposed to the proteomes of WT animals on *S. enterica* where several of the same proteins were upregulated during infection. These results suggest that loss of NMUR-1 function inhibits transmembrane transport and “ATP synthesis coupled proton transport,” which may contribute to the enhanced survival in *S. enterica*-infected *nmur-1(ok1387*) animals. During *S. enterica* pathogenesis, the bacteria cause an increase in host ROS production, leading to cellular damage (19, 24–26). ATP production is an important source of ROS, and hindering ATP synthesis reduces ROS production, which, in turn, would slow *S. enterica* pathogenesis (27–30). The decrease in the abundance of ATP synthesis proteins and presumably ROS in the *nmur-1(ok1387*) animals provides a possible mechanism responsible for their increased survival against *S. enterica*.

We next compared the proteomes of *nmur-1(ok1387*) animals and WT animals during *E. faecalis* infection and found that 85 and 221 proteins were upregulated and downregulated, respectively. Although a GO enrichment analysis on the 85 upregulated proteins yielded no significantly enriched processes, a GO analysis on the 221 downregulated proteins identified 18 enriched biological processes. Again, the most significantly downregulated group was “Transmembrane transport” (q-value = 4.96E-7). Its subgroup, “ATP synthesis-coupled proton transport,” was also found to be significantly downregulated (q-value = 5.38E-3) (Fig. 2B). The downregulation of ATP synthesis-related proteins may contribute to the reduced survival of *nmur-1(ok1387*) animals against *E. faecalis*. It has been shown that *E. faecalis* can colonize the intestine of *C. elegans* (16) and that the production of a specific ROS, H_2_O_2_, is required for survival (31, 32). During *E. faecalis* infection, *C. elegans* expresses BLI-3 that utilizes NADP(H) to generate H_2_O_2_ (31). Since generating NADP(H) is an ATP-dependent process, a reduction in ATP availability would reduce the levels of NADPH and H_2_O_2_ (21, 31, 33–35). The lower abundance of ATP synthesis proteins and available ATP in *nmur-1(ok1387*) animals during *E. faecalis* infection could slow H_2_O_2_ production and lead to reduced survival.

To confirm the above-described findings, we repeated the GO analysis using the *C. elegans*-specific enrichment tools on WormBase (https://wormbase.org//tools/enrichment/tea/tea.cgi), which produced highly consistent results, again highlighting proteins involved in cellular respiration and the electron transport chain as the most enriched categories (Fig. S1). Proteomic differences between WT and *nmur-1(ok1387*) animals were validated by western blot analysis. We focused on two proteins of interest, HSP-60 and ATP-4, both of which were found to be downregulated in *nmur-1(ok1387*) animals during infection in the proteomic data. Western blots using whole-animal lysates from *S. enterica*- or *E. faecalis*-infected animals confirmed the reduced expression of HSP-60 and ATP-4 in *nmur-1(ok1387*) mutants compared with WT controls (Fig. S2A). Densitometric quantification of normalized band intensities further supported these findings, showing decreased levels of both proteins in the mutants (Fig. S2B). To confirm antibody specificity, we validated HSP-60 and ATP-4 antibodies by RNAi-mediated knockdown, which resulted in marked reduction of their respective protein signals compared with controls (Fig. S3).

Overall, *nmur-1(ok1387*) animals exhibited significantly reduced expression of proteins involved in transmembrane transport and ATP synthesis during infection with either *S. enterica* or *E. faecalis*. Given the essential role of ATP production in maintaining cellular energy homeostasis, impaired ATP synthesis may contribute to the divergent survival outcomes observed in *nmur-1(ok1387*) animals in response to these two pathogens, potentially through distinct mechanisms.

### Altered expression of ATP synthesis proteins influences *C. elegans* survival during *S. enterica* and *E. faecalis* infections

To explore whether a lack of ATP synthesis proteins contributes to the survival phenotypes of *nmur-1(ok1387*) animals, we compared the proteins downregulated in both *S. enterica* and *E. faecalis* pathogenic conditions that fell within the same GO group “ATP synthesis-coupled proton transport” to identify shared proteins. Seven proteins were identified as shared between the different conditions: ATP-3, ATP-4, ATP-5, ASB-1, ASB-2, R53.4, and COX-5B. Five of the seven proteins were predicted subunits of the F_1_F_O_ ATP synthase complex: ATP-3, ATP-4, ATP-5, ASB-1, and ASB-2. We narrowed our focus to ATP-3, ATP-4, and ATP-5, as these three proteins are subunits of the F_O_ peripheral stalk, which acts as a stator for the F_1_ catalytic head, and are homologs to human ATP5PD, ATP5PO, and ATP5PF, respectively (36–38).

**Fig 3:**
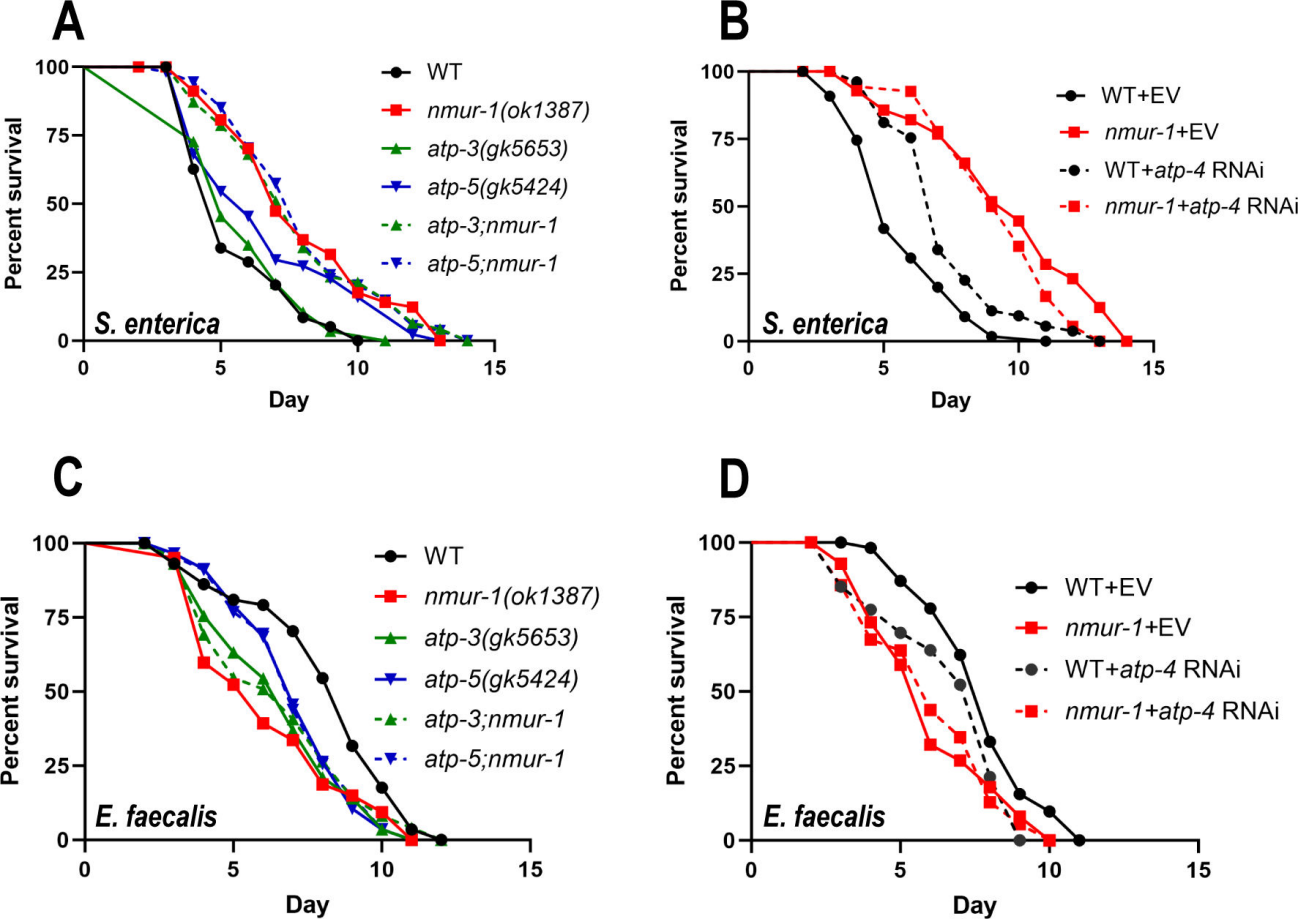
NMUR-1-regulated F_1_F_O_ ATP synthase subunits are required for defense against *S. enterica* and *E. faecalis*. (**A**) WT, *nmur-1(ok1387), atp-3(gk5653), atp-5(gk5424),* and *atp-3(gk5653);nmur-1(ok1387),* and *atp-5(gk5424);nmur-1(ok1387*) animals were exposed to *S. enterica* and scored for survival over time. The graphs are a representation of three independent replicates. Each experiment included 60 animals per strain. *P* values of mutants relative to WT: *nmur-1(ok1387), P* < 0.0001; *atp-3(gk5653)*, *P* = 0.8581; and *atp-5(gk5424)*, *P* = 0.0053. *P* values of double mutants relative to *nmur-1(ok1387): atp-3(gk5653);nmur-1(ok1387)*, *P* = 0.8736; *atp-5(gk5424);nmur-1(ok1387)*, *P* = 0.9711 . (**B**) WT and *nmur-1(ok1387)* animals grown on dsRNA for *atp-4* or the empty vector (EV) control were exposed to *S. enterica* and scored for survival over time. The graphs are a representation of three independent replicates. Each experiment included *n* = 60 animals per strain. *P* values of RNAi treatments relative to WT + EV: *nmur-1(ok1387)* + EV, *P* < 0.0001; WT + *atp-4*, *P* < 0.0001. *P* value of RNAi treatment relative to *nmur-1(ok1387)* + EV: *nmur-1(ok1387) + atp-4*, *P* = 0.0939. (**C**) WT, *nmur-1(ok1387), atp-3(gk5653), atp-5(gk5424), atp-3(gk5653);nmur-1(ok1387),* and *atp-5(gk5424);nmur-1(ok1387)* animals were exposed to *E. faecalis* and scored for survival over time. The graphs are a representation of three independent replicates. Each experiment included *n* = 60 animals per strain. *P* values of mutants relative to WT: *nmur-1(ok1387), P* < 0.0001; *atp-3(gk5653)*, *P* = 0.0002; *atp-5(gk5424)*, *P* = 0.0011. *P* values of double mutants relative to *nmur-1(ok1387): atp-3(gk5653);nmur-1(ok1387)*, *P* = 0.4323; *atp-5(gk5424);nmur-1(ok1387)*, *P* = 0.1924. (**D**) WT and *nmur-1(ok1387)* animals grown on dsRNA for *atp-4* or EV were exposed to *E. faecalis* and scored for survival over time. The graphs are a representation of three independent replicates. Each experiment included *n* = 60 animals per strain. *P* values of RNAi treatments relative to WT + EV: *nmur-1(ok1387)* + EV, *P* = 0.0001; WT + *atp-4*, *P* = 0.0113. *P* values of RNAi treatment relative to *nmur-1(ok1387)* + EV: *nmur-1(ok1387) + atp-4*, *P* = 0.8933.

First, we sought to determine if inactivation of individual ATP synthase subunits has any influence on worm survival against *S. enterica*. To this end, we crossed *atp-3(gk5653*) or *atp-5(gk5424*) knockout mutant animals with *nmur-1(ok1387*) animals to generate double mutants. We also silenced *atp-4* via RNA interference (RNAi) in both WT and *nmur-1(ok1387*) mutant animals (16). Although inactivation of these genes displayed various survival phenotypes in the WT background (*atp-3(gk5653*) animals showed WT survival, and *atp-5(gk5424*) and *atp-4* RNAi animals showed enhanced survival), *nmur-1(ok1387*) animals with such gene inactivation did not display any significant changes in survival time (Fig. 3A and B), indicating that NMUR-1 and these ATP synthesis subunits might function in the same pathway to influence worm defense and survival against *S. enterica*.

We also examined the roles of individual ATP synthase subunits in worm survival against *E. faecalis*. Our results showed that *atp-3(gk5653)* and *atp-5(gk5424)* mutant animals displayed reduced survival against *E. faecalis* infection; however, *nmur-1(ok1387)* mutant animals carrying the same *atp-3* or *atp-5* mutation showed no significant difference in survival compared with *nmur-1(ok1387*) single mutants (Fig. 3C). Silencing *atp-4* also significantly reduced the survival of WT animals against *E. faecalis* infection but did not significantly affect that of *nmur-1(ok1387)* animals (Fig. 3D). Taken together, although inactivation of *atp-3, atp-4*, or *atp-5* affected the survival of WT animals during *E. faecalis* infection, the survival of *nmur-1(ok1387)* mutant animals was unaffected by the loss of either of these genes against the same pathogen. These results suggest that the ATP synthesis pathway is critical for defense against *S. enterica* and *E. faecalis* and that this pathway is suppressed in *nmur-1* mutants.

**Fig 4:**
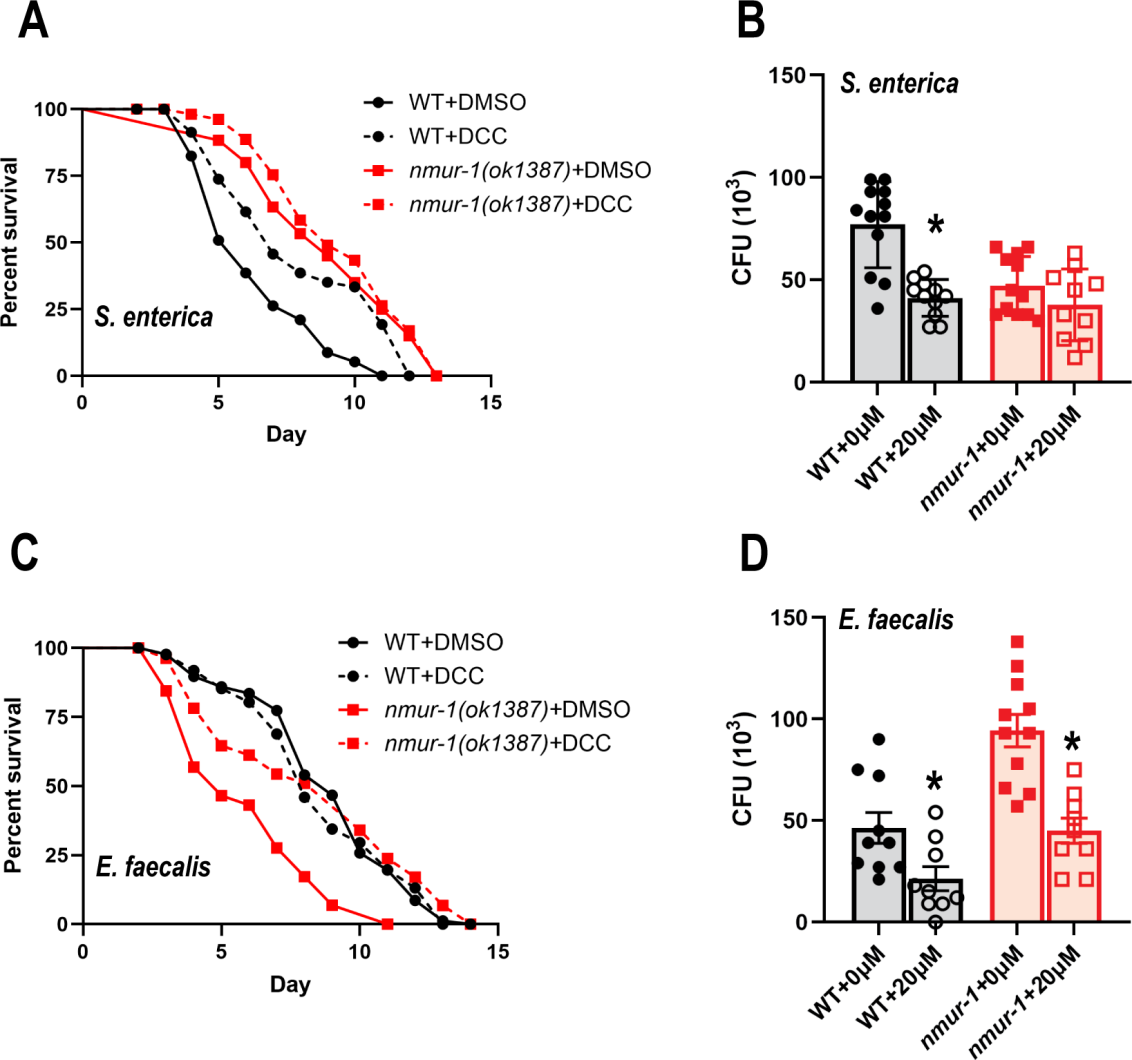
Chemical inhibition of F_1_F_O_ ATP synthase increased the survival of WT and *nmur-1(ok1387)* animals against *S. enterica* and *E. faecalis*, respectively. (**A, C**) WT and *nmur-1(ok1387)* animals were exposed to *S. enterica* (**A**) or *E. faecalis* (**C**) on plates containing either DMSO or 20 µM of DCC and scored for survival over time. Each graph is a representative of three independent experiments. Each experiment included *n* = 60 animals per strain. *P* values represent the significance levels of DCC treatment relative to DMSO: in (**A**), WT + DCC vs. WT + DMSO, *P* < 0.0001; *nmur-1(ok1387)* + DCC vs. *nmur-1(ok1387)* + DMSO, *P* = 0.4660; in (**C**), WT + DCC vs. WT + DMSO, *P* = 0.7625; *nmur-1(ok1387)* + DCC vs. *nmur-1(ok1387)* + DMSO, *P* < 0.0001. (**B, D**) WT and *nmur-1(ok1387)* animals were exposed to *S. enterica* (**B**) or *E. faecalis* (**D**) on plates containing either DMSO or 20 µM of DCC for 24 h, and the CFUs of live bacterial cells were recovered from the intestines. The graph represents the combination of three independent experiments, *n* = 10 animals per experiment. Error bars represent the SEM. *P* values represent the significance levels of DCC treatment relative to DMSO: in (**B**), WT + 20 µM vs. WT + 0 µM, *P* < 0.0001; *nmur-1(ok1387)* + 20 µM vs*. nmur-1(ok1387)* + 0 µM, *P* = 0.1906; in (**D**), WT + 20 µM vs. WT + 0 µM, *P* < 0.0202; *nmur-1(ok1387)* + 20 µM vs*. nmur-1(ok1387)* + 0 µM, *P* = 0.0002.

To investigate whether disrupting the function of F_1_F_O_ ATP synthase has an influence on worm survival during *S. enterica* or *E. faecalis* infections, we treated the animals with *N,N'*-Dicyclohexylcarbodiimide (DCC) to chemically inhibit ATP synthesis. DCC is a classic inhibitor of ATP synthesis and functions by binding to the F_O_ subunit, inducing steric hindrance and preventing rotation of the F_O_ c-ring subunit required for ATP synthesis (39, 40). WT animals exposed to DCC during *S. enterica* infection had a significant increase in survival compared with the vehicle DMSO control (Fig. 4A), likely because the pathogenesis of *S. enterica* relies on an intrinsic induction of ROS in *C. elegans* that depends on ATP production (19)*.* However, the *nmur-1(ok1387)* mutants had no difference in survival against *S. enterica* between the DCC and vehicle control treatments (Fig. 4A), possibly due to the already downregulated key F_1_F_O_ ATP synthase subunits composing the peripheral stalk in the mutants (Fig. 2A). The peripheral stalk works in tandem with the membrane-bound F_O_ c-ring to convert the flow of protons across the mitochondrial membrane into ATP (41, 42). Without the peripheral stalk, ATP production by F_1_F_O_ ATP synthase is hindered, and inhibition of the c-ring by DCC does not further inhibit ATP production. Thus, the lack of key components forming the F_1_F_O_ ATP synthase peripheral stalk likely made the *nmur-1(ok138)* mutant animals resistant to DCC treatment. Together, these results indicate that downregulation of F_1_F_O_ ATP synthase in *nmur-1(ok1387)* animals may cause a decrease in ATP production, which likely leads to an enhanced survival of *nmur-1(ok1387)* mutant animals against *S. enterica*.

To investigate whether DCC affects *S. enterica* in the context of host-pathogen interaction, we performed colony-forming unit (CFU) assays to quantify bacterial colonization in the *C. elegans* intestine. Our results revealed that WT animals infected with *S. enterica* and treated with DCC had significantly reduced intestinal bacterial loads compared with untreated controls (Fig. 4B). In contrast, *nmur-1(ok1387)* mutants showed no significant change in colonization between DCC and vehicle DMSO treatments (Fig. 4B). These findings correlate with our survival data: DCC increased the survival in WT animals during *S. enterica* infection but had no significant effect on *nmur-1(ok1387)* mutants (Fig. 4A). This is likely due to the downregulation of key F₁F₀ ATP synthase subunits in *nmur-1* mutants, which renders them less sensitive to further ATP synthase inhibition.

Exposure to DCC, however, significantly increased the survival of *nmur-1(ok1387)* mutants against *E. faecalis* relative to the DMSO control*,* but WT animals showed no significant difference between DCC treatment and the vehicle DMSO control (Fig. 4C). The increased survival in *nmur-1(ok1387)* animals with DCC treatment was unexpected and opposite to the above-described result with *S. enterica* infection. Our CFU assays demonstrated that DCC treatment reduced the intestinal colonization of *E. faecalis* in both WT and *nmur-1(ok1387)* mutant animals (Fig. 4D). A possible explanation for these results is that DCC has antimicrobial properties, as the chemical can inhibit both eukaryotic and prokaryotic F_1_F_O_ ATP synthase. When *E. faecalis* is exposed to DCC, the bacteria display a greater retention of the antibiotic gentamicin present in the media; this is because the exportation of gentamicin from bacterial cells requires ATP (43, 44). Inhibition of the bacterial ATP synthase by DCC results in increased intracellular antibiotic retention and reduced bacterial viability. This mechanism likely accounts for the observed reduction in colonization following DCC treatment in both WT and *nmur-1(ok1387)* animals. We were unable to perform worm survival assays against *E. faecalis* without the use of an antibiotic, as it was necessary to eliminate residual non-pathogenic *E. coli*, the worms' standard food source, which can become pathogenic when grown on the BHI medium used in the *E. faecalis* survival assays (45). Our current CFU assays (Fig. 4D), together with our previous work (16), show that without DCC treatment, *nmur-1(ok1387)* mutants exhibit significantly higher *E. faecalis* colonization compared with WT animals, which correlates with the mutants’ reduced survival against this pathogen. This provides a mechanistic explanation for how DCC treatment rescues the survival phenotype in *nmur-1(ok1387)* animals by lowering the bacterial burden. In contrast, DCC treatment did not significantly improve WT animals’ survival. This may be due to the non-linear relationship between pathogen load and host survival in *C. elegans*, where survival only improves once bacterial colonization falls between certain thresholds (1).

To further investigate whether DCC impacts bacterial viability, we performed growth curve assays for both *S. enterica* and *E. faecalis* in the presence and absence of DCC. Our data revealed that DCC had no measurable effect on the growth rate of *S. enterica* but significantly slowed the growth of *E. faecalis* (Fig. S4), supporting our interpretation that DCC exerts a direct antimicrobial effect on *E. faecalis* but not on *S. enterica*.

### Loss of NMUR-1 function reduces ATP concentrations during pathogen infection

**Fig 5:**
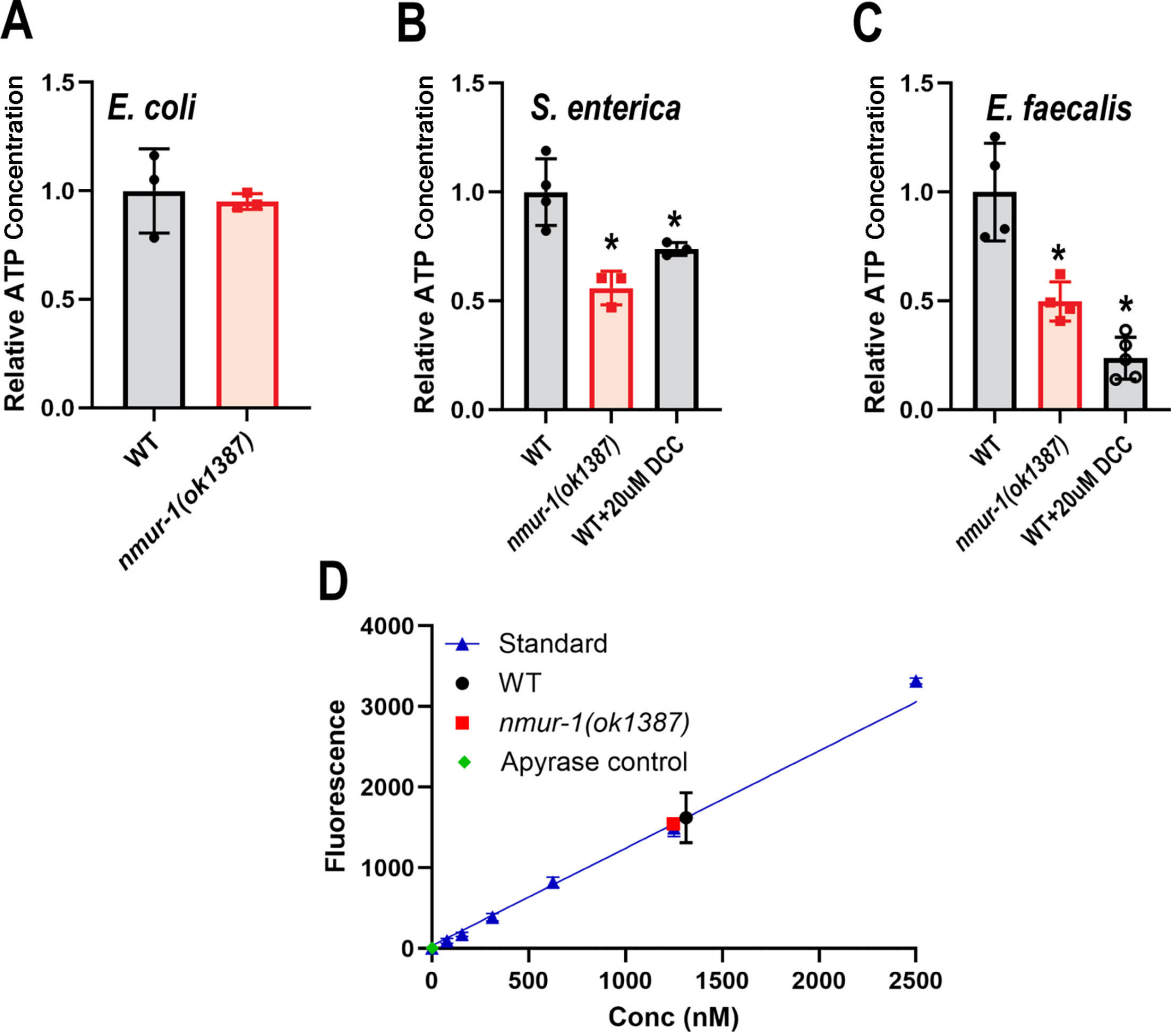
NMUR-1 regulates ATP levels during pathogen infection. (A–C) Relative ATP concentrations in WT and *nmur-1(ok1387)* animals exposed to *E. coli* (**A**), *S. enterica* (**B**), or *E. faecalis* (**C**) for 24 h, with or without 20 µM DCC treatment. ATP concentrations are normalized to uninfected (in Panel A) or infected (in Panels B and C) WT animals without DCC treatment. The data represent the combined results of three independent experiments. In each experiment, 30 animals of each strain under each condition were used. Error bars represent standard deviation. Asterisk (*) denotes a significant difference (*P* < 0.05) between mutants or treatments and WT controls, as analyzed using Dunnett’s multiple comparison test. (**D**) Dynamic range of the ATP assay. ATP concentrations from WT and *nmur-1(ok1387)* animals exposed to *E. coli* were plotted against a standard ATP titration curve (0−5,000 nM). Apyrase-treated lysates from both strains served as negative controls, confirming complete hydrolysis of endogenous ATP. Measured ATP concentrations were ~1,314 nM in WT and ~1,137 nM in *nmur-1(ok1387)* animals, values within the linear range of the standard curve.

Next, we examined ATP concentrations in WT and *nmur-1(ok1387)* animals during *S. enterica* and *E. faecalis* infections using a luminescence method (46). We first measured the levels of ATP in both WT and *nmur-1(ok1387)* animals under non-pathogenic conditions (grown on *E. coli* HT115), and our results showed that WT and *nmur-1(ok1387)* animals had similar ATP concentrations (Fig. 5A). However, during *S. enterica* infection, *nmur-1(ok1387)* animals had significantly lower ATP concentrations compared with WT animals (Fig. 5B). Decreased ATP concentrations were also observed in *nmur-1(ok1387)* animals during *E. faecalis* infection (Fig. 5C). Interestingly, ATP concentrations in animals infected with *S. enterica* were significantly higher than in those infected with *E. faecalis* (approximately 3-fold higher in both WT and *nmur-1(ok1387)* animals) (Fig. S5). This finding aligns with previous studies demonstrating that *S. enterica* actively induces host ATP production for its pathogenic ROS generation (19), whereas *E. faecalis* infection prompts the host to consume ATP to produce H_2_O_2_ as a defense mechanism (31). As a control, we quantified the ATP concentrations in WT animals exposed to either *S. enterica* or *E. faecalis* after being treated with DCC using the same method (DCC inhibits ATP synthesis and should reduce ATP concentrations). Comparing the ATP concentrations in animals exposed to either 20 µM DCC or the vehicle DMSO control, animals exposed to DCC had significantly reduced ATP concentrations after 24 h (Fig. 5B and C). This validates our ATP quantification method and also confirms the efficiency of DCC in inhibiting ATP production during a 24 h pathogen exposure. To validate the dynamic range of our ATP assay, we compared lysates from WT and *nmur-1(ok1387)* animals exposed to *E. coli* against a standard ATP titration curve and included apyrase-treated lysates as negative controls. Both WT and *nmur-1(ok1387)* ATP concentrations (~1,314 nM and ~1,137 nM, respectively) fell within the linear range of the assay (Fig. 5D). Taken together, these results demonstrate that loss of NMUR-1 function reduces ATP concentrations during pathogen infection, which may contribute to the survival phenotypes of *nmur-1(ok1387)* animals against *S. enterica* and *E. faecalis*.

### Loss of NMUR-1 function reduces ROS concentrations during pathogen infection

**Fig 6:**
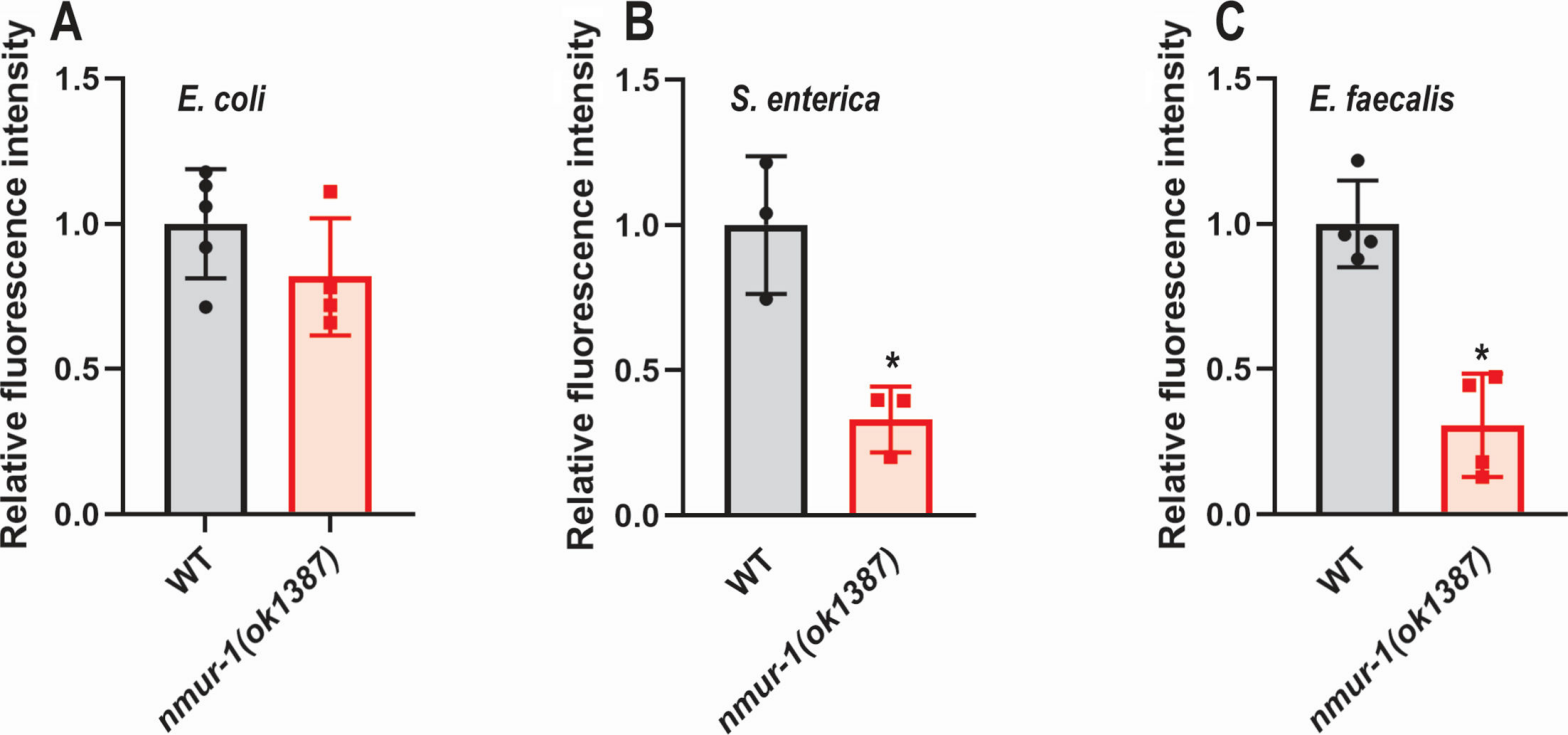
ROS concentrations were lower in *nmur-1(ok1387)* animals than in WT animals exposed to pathogens. WT and *nmur-1(ok1387)* animals were exposed to *E. coli* (**A**), *S. enterica* (**B**) or *E. faecalis* (**C**) for 24 h. The animals were stained for ROS production using H_2_DCFDA, and fluorescence was monitored every 30 min for 6 h. A representative timepoint between 3 and 4 h, when reproducible differences between strains first became evident, was selected for analysis. The graphs show the combined results of three independent replicate experiments, which measured the ROS production of 30 animals per replicate. The results are relative to the normalized fluorescence in WT animals, and an asterisk (*) denotes a significant difference (*P*  <  0.05) as determined by Student’s *t*-test.

Next, we sought to determine if ROS concentrations were also affected by the reduction in ATP availability in *nmur-1(ok1387)* mutant animals. To determine ROS concentrations in animals infected with either *S. enterica* or *E. faecalis*, we followed a previously established protocol utilizing Dichlorodihydrofluorescein Diacetate (47). WT and *nmur-1(ok1387)* mutant animals were exposed to *E. coli*, *S. enterica,* or *E. faecalis* for 24 h prior to ROS quantification. Compared with WT animals, *nmur-1(ok1387)* animals did not display any significant difference in ROS concentration when exposed to *E. coli* (Fig. 6A). However, during either *S. enterica* or *E. faecalis* infection, *nmur-1(ok1387)* mutant animals had a significantly lower fluorescent intensity, correlating to a lower ROS concentration (Fig. 6B and C). These findings support our previous notion that lower ATP availability may lead to lower ROS concentrations in *nmur-1(ok1387)* mutant animals. Overall, these results are consistent with previous studies highlighting the importance of ROS during *S. enterica* and *E. faecalis* infection, and *nmur-1* plays a significant role in regulating its concentration.

### The NMUR-1 endogenous ligand CAPA-1 and its expressing neurons ASG regulate ATP levels during *E. faecalis* infection

**Fig 7:**
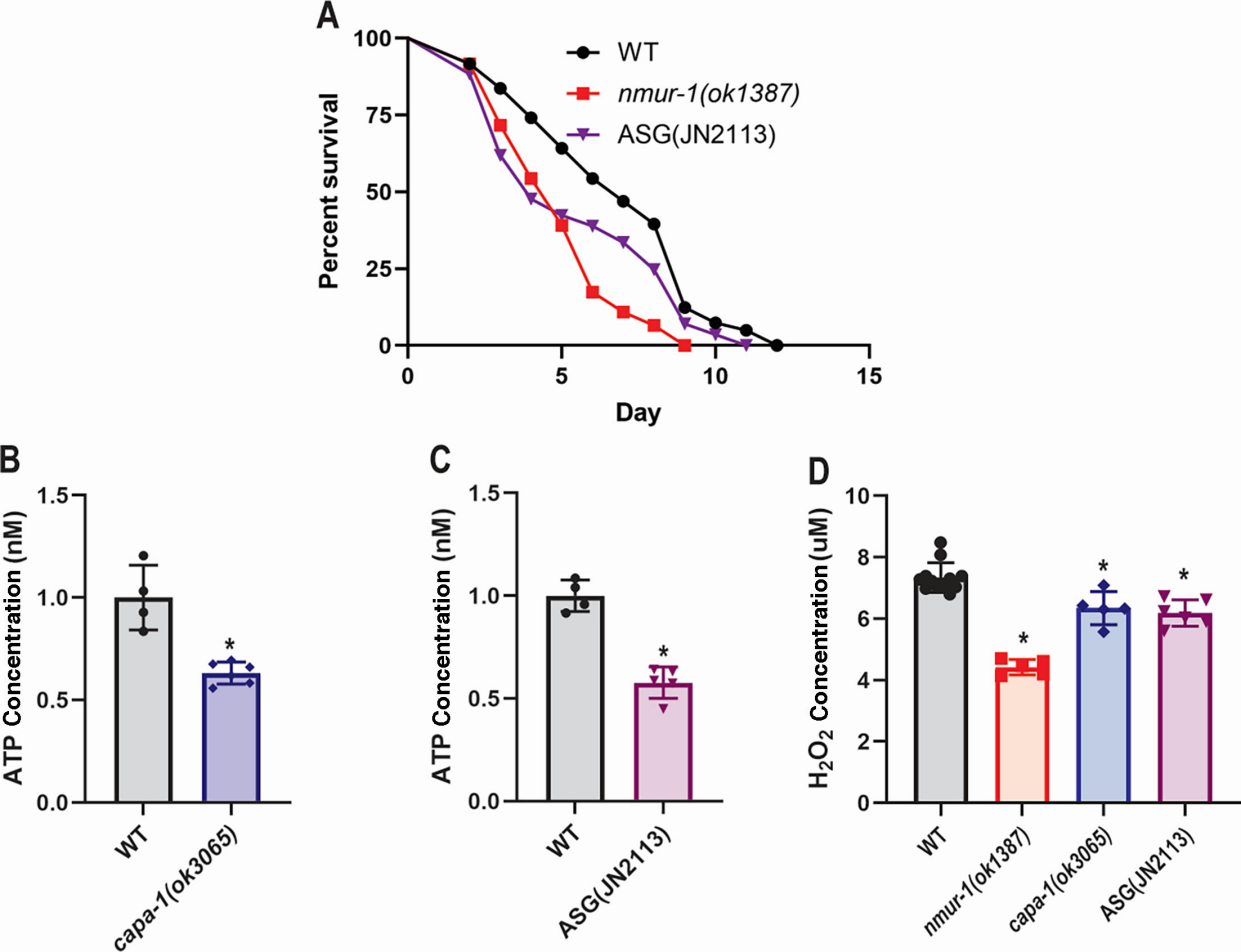
Loss of CAPA-1 or ASG neurons partially mimicked the phenotypes of *nmur-1(ok1387)* animals against *E. faecalis*. (**A**) WT, *nmur-1(ok1387)*, and ASG ablated JN2113 animals were exposed to *E. faecalis* and scored for survival over time. The graphs are a representation of three independent replicates. Each experiment included 60 animals per strain. *P* values represent the significance levels of mutants relative to WT: *nmur-1(ok1387), P* < 0.0001; JN2113, *P* = 0.0292. (**B-C**) WT, *capa-1(ok3065)*, and JN2113 animals were exposed to *E. faecalis* for 24 h, followed by ATP concentration measurements. The graphs show the combined results of three independent experiments. In each experiment, 30 animals of each strain were used. Error bars represent standard deviation. Asterisk (*) denotes a significant difference (*P*  <  0.05) between mutants and WT animals. (**D**) Animals were exposed to *E. faecalis* for 24 h. H_2_O_2_ concentrations were quantified using Amplex Red fluorescent dye after a 30 min incubation. The graphs show combined results of three independent replicates, which measured the H_2_O_2_ production of 30 animals per replicate. The results were normalized to the number of animals collected per experiment. Error bars represent standard deviation. Asterisk (*) denotes a significant difference (*P*  <  0.05) between treatment and the untreated WT control, as determined by Dunnett’s multiple comparison test.

Our previous research revealed that the NMUR-1 endogenous ligand CAPA-1 expressed by the ASG neurons was required for the survival of *C. elegans* against *E. faecalis* but dispensable in defense against *S. enterica* (16). Here, we focused on investigating whether the ATP level is regulated by the NMUR-1/CAPA-1/ASG signaling in the context of *E. faecalis* infection. The knockout mutant *capa-1(ok3065)* displays a reduced survival phenotype against *E. faecalis* similar to *nmur-1(ok1387)* (16) (Fig. S6). Since CAPA-1 is only expressed in a pair of ASG amphid neurons (48), we questioned if the removal of these neurons would also have a similar effect on survival against *E. faecalis*. Measuring the survival of an ASG-ablated strain against *E. faecalis,* we found the lack of ASG neurons indeed led to reduced survival (Fig. 7A). Quantifying ATP concentrations in both *capa-1(ok3065)* knockout animals and ASG-ablated animals during *E. faecalis* infection found that both strains had significantly reduced ATP levels compared with WT animals (Fig. 7B and C). We also measured H₂O₂ levels following *E. faecalis* infection across all four strains. As previously described, BLI-3-mediated H₂O₂ production is an ATP-dependent component of *C. elegans* defense against *E. faecalis*. We found that *nmur-1(ok1387)*, *capa-1(ok3065)*, and ASG-ablated strains produced significantly lower levels of H₂O₂ compared with WT animals (Fig. 7D). Notably, the reduction was most pronounced in *nmur-1* mutants, suggesting that NMUR-1 signaling plays a key role in ROS production. The more modest decreases observed in *capa-1* mutants and ASG-ablated animals suggest that CAPA-1 and ASG neurons contribute partially to this response. These results indicate that the CAPA-1/NMUR-1/ASG signaling regulates the ATP level in the context of *E. faecalis* infection, which likely contributes to the overall defense against this pathogen via ROS production.

### Our current proteomic data and previous transcriptomic data reveal different aspects of NMUR-1-mediated host defense against pathogen infection

**Fig 8:**
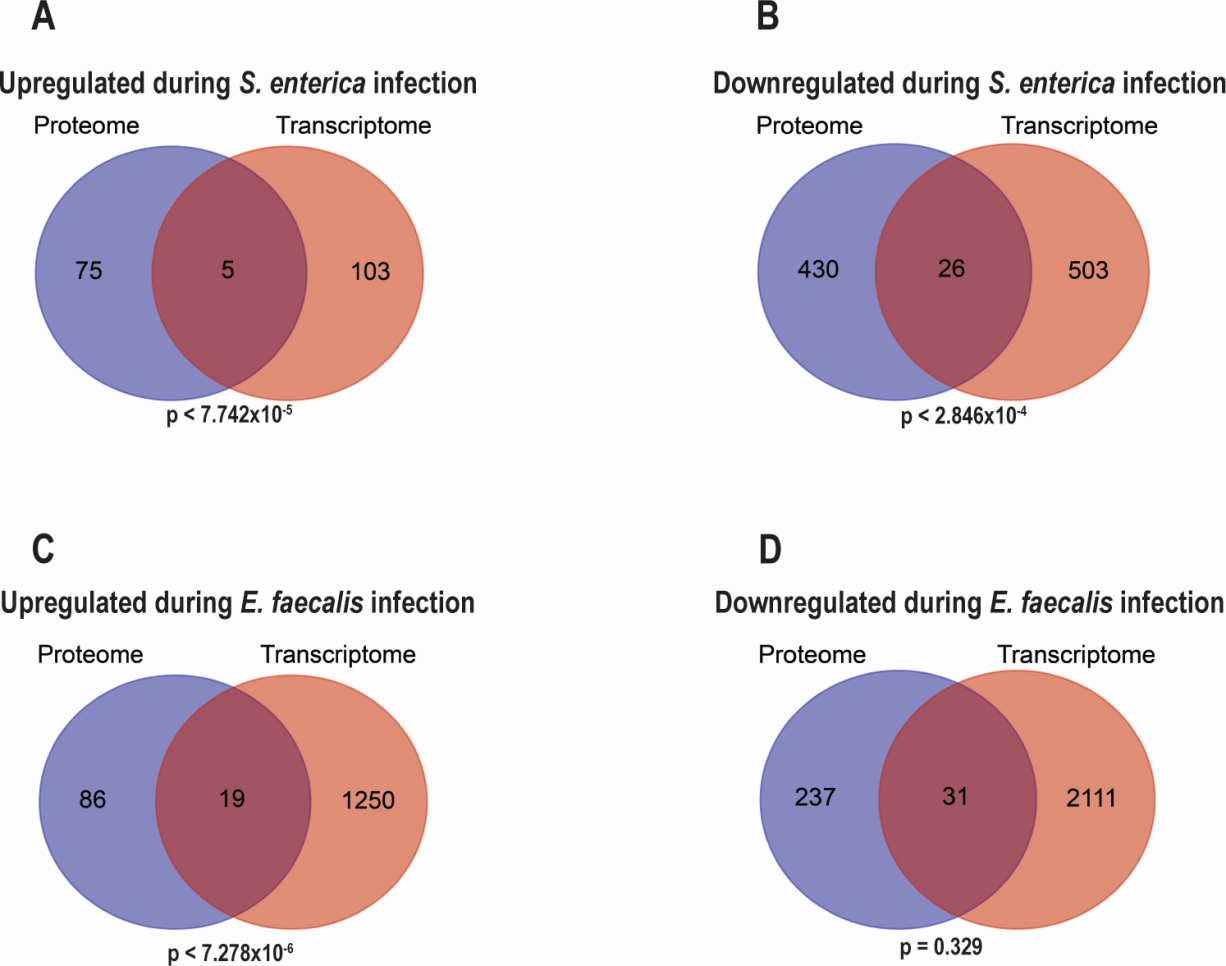
Venn diagrams show the overlaps between differentially regulated genes and proteins in our transcriptomic and proteomic data. Significantly upregulated or downregulated genes and proteins (≥1.5-fold) in *nmur-1(ok1387)* animals relative to WT animals during infection with *S. enterica* (**A and B**) or *E. faecalis* (**C and D**) were compared to identify overlapping genes and proteins (the transcriptomic data can be accessed through GEO with the accession number GSE154324; the proteomic data can be accessed through PRIDE with the accession number PXD045826). The statistical significance of the overlap between two lists was calculated based on a hypergeometric distribution (49).

Previously, we have performed a transcriptomic study to profile gene expression changes in *nmur-1(ok1387)* animals relative to WT animals under *S. enterica* and *E. faecalis* infections (16). To determine whether these transcriptomic data are concordant with our current proteomic data, we compared the two datasets at both the individual gene/protein level and the GO term level. At the individual gene/protein level, we identified 14,363 genes and 2,968 proteins (at 5% FDR) in *nmur-1(ok1387)* animals relative to WT animals exposed to *S. enterica*, reflecting nearly a 5-fold difference in detection sensitivity between the transcriptomic and proteomic technologies used. Among these, 103 genes and 75 proteins were significantly upregulated (≥1.5 fold), with 5 of them overlapping. This overlap is statistically significant (*P* < 7.742×10^−5^) (Fig. 8A). Similarly, 503 genes and 430 proteins were significantly downregulated (≥1.5-fold), with 26 of them overlapping, also statistically significant (*P* < 2.846 × 10^−4^) (Fig. 8B). Under *E. faecalis* infection, 15,064 genes and 2,947 proteins were identified at 5% FDR. Of these, 1,250 genes and 86 proteins were significantly upregulated (≥1.5-fold), with 19 of them overlapping. This overlap is statistically significant (*P* < 7.278 × 10^−6^) (Fig. 8C). In contrast, although 2,111 genes and 237 proteins were significantly downregulated, only 31 overlapped, and this overlap was not statistically significant (*P* = 0.329) (Fig. 8D). A detailed list of the overlapping genes and proteins is provided in Table S1. A further examination of these overlaps reveals that many of them are highly expressed and involved in multiple tissues, including muscle, neurons, intestine, and cephalic sheath cells (WormBase). Their abundance likely accounts for their detection by both RNA-seq and mass spectrometry. Moreover, GO analyses of these overlapping genes/proteins reveal that they are enriched in categories related to signaling, metabolism, immune response, and energy production/electron transport (Fig. S7). Thus, these overlaps represent highly expressed, functionally important genes/proteins involved in defense responses to pathogen infection. Overall, our comparative analyses uncover statistically significant overlaps between the transcriptome and proteome data sets, except for the downregulated group under *E. faecalis* infection.

At the GO term level, although the transcriptomic analyses revealed the top enriched GO terms involve unfolded protein responses and carbohydrate binding during *S. enterica* and *E. faecalis* infections, respectively (16), the top enriched GO terms derived from proteomic analyses are centered at mitochondrial transmembrane transport and ATP synthesis during both *S. enterica* and *E. faecalis* infections (Fig. 2). These findings demonstrate that the transcriptomic and proteomic data sets are discordant at the GO term level, despite their largely significant overlaps in individual genes and proteins. This observation is consistent with recent research reporting poor correlations between mRNA and protein expression levels (50–54). Several factors could contribute to such a discordance, including technological biases leading to a much lower proteome coverage than transcriptome coverage (e.g., 5-fold differences in the numbers of genes and proteins detected in our case) and multiple regulatory layers such as post-transcriptional regulation, differences in translation efficiency, post-translational modifications, protein degradation rates, and additional mechanisms that control protein abundance independently of mRNA levels (50–54). Because of these reasons, we propose that our transcriptomic and proteomic studies are complementary to each other, uncovering different aspects of NMUR-1-mediated host defense against pathogen infection.

## DISCUSSION

In our current study, we have shown that *C. elegans* lacking the neuronal GPCR NMUR-1 have an altered proteome when challenged with the pathogen *S. enterica* or *E. faecalis*. We focused our attention on these two pathogens, as *nmur-1* mutant animals display opposing survival phenotypes when infected with these pathogens, which can serve as a useful model for understanding the roles of NMUR-1 in mediating the specificity of innate immune responses (16). Utilizing quantitative mass spectrometry, we found that animals with an *nmur-1* knockout have a significant decrease in the expression of proteins responsible for ATP biosynthesis during infection. This is interesting as studies have already pointed to the role of ATP biosynthesis and the mitochondria in regulating the innate and adaptive immune responses (55–57). We hypothesized that this decrease in ATP biosynthesis proteins plays a role in the survival phenotypes of *nmur-1(ok1387)* mutant animals challenged with *S. enterica* and *E. faecalis*. Indeed, inhibition of ATP synthesis caused WT animals to partially mimic the survival of *nmur-1(ok1387)* mutant animals during *S. enterica* or *E. faecalis* infection. We also questioned if specific neurons played any role in regulating ATP production through an NMUR-1-dependent pathway. Our previous studies have shown that the endogenous ligand CAPA-1 was required for survival against *E. faecalis,* and other labs have shown that CAPA-1 is expressed in the ASG neurons (16, 48). Indeed, we found that CAPA-1 and the ASG neurons are required for NMUR-1-mediated *C. elegans* defense against *E. faecalis,* and a lack of either causes a decrease in ATP level (Fig. 7). We noted the incomplete phenotypic recapitulation of H_2_O_2_ concentrations in *capa-1(ok3065)* mutants or ASG-ablated animals (Fig. 7D), which suggests that additional ligands may contribute to NMUR-1 activation. Indeed, *E. faecalis* produces bioactive peptides (e.g., gelatinase-cleaved products [58]) and quorum-sensing molecules (e.g., peptide pheromones [59]) that could potentially modulate host GPCR signaling. Future studies should test whether *E. faecalis*-secreted peptides directly activate NMUR-1 or the residual ATP/ROS in *capa-1* mutants reflects NMUR-1-independent compensatory pathways.

Previously, we have performed a transcriptomic study to profile gene expression changes in *nmur-1(ok1387)* animals relative to WT animals under *S. enterica* and *E. faecalis* infections (16). That study and the current proteomic study were conducted under similar conditions, and there are significant overlaps between differentially expressed genes and proteins in the transcriptomic and proteomic data sets, except for the downregulated group under *E. faecalis* infection (Fig. 8). However, the transcriptomic and proteomic data sets are largely discordant based on the GO enrichment analyses in the two studies. This observation is consistent with recent research reporting poor correlations between mRNA and protein expression levels (50–54). Several factors could contribute to this discordance: (ⅰ) technological biases lead to a much lower proteome coverage than transcriptome coverage, (ⅱ) post-transcriptional regulation plays a significant role, (ⅲ) translation efficiency varies among different mRNAs, (ⅳ) post-translational modifications are crucial for protein function, (ⅴ) protein stability and degradation significantly affect protein abundance, and (ⅵ) gene expression regulation involves mechanisms like microRNAs, RNA-binding proteins and ribosomal proteins, which regulate how much protein is synthesized from a given mRNA. Therefore, the overall discordance between the transcriptomic and proteomic responses in *nmur-1* mutants is expected due to intrinsic differences between the two molecular layers.

How is NMUR-1, a single neuronal GPCR, able to regulate ATP levels in response to various pathogens but mediate differing survival phenotypes? During *E. faecalis* infection, ASG neurons likely secrete CAPA-1 that may bind to NMUR-1 and increase ATP and ROS availability. This signaling cascade promotes the survival of *C. elegans* against *E. faecalis*. In the case of *S. enterica* infection, however, animals lacking CAPA-1 do not share an enhanced resistant phenotype like NMUR-1 knockout animals (16). This points to the possibility that upon *S. enterica* infection, *C. elegans* is not activating NMUR-1 via CAPA-1 and triggering the downstream increase in ATP and ROS levels. Instead, *S. enterica* may produce an exogenous ligand that binds to NMUR-1. This is supported by another study investigating how *S. enterica* is able to colonize the intestine of *C. elegans* (19)*.* The authors found that animals infected with *S. enterica* had an increased concentration of ROS during infection and that this increase in ROS was necessary for killing by *S. enterica* (19). In *nmur-1(ok1387)* mutants, a reduction in ATP synthase expression (Fig. 2A) prevents *S. enterica*-induced increase in mitochondrial ROS (Fig. 6B). Because *S. enterica* pathogenesis relies on host-derived ROS to cause cellular damage (19), this disruption in ROS generation enhances host resistance. This dual role as both a receptor for mounting a proper immune response against *E. faecalis* and a vulnerability against *S. enterica* places NMUR-1 in a pivotal space in host-pathogen interactions. We noted that there were no differences in ATP or ROS concentrations in uninfected *nmur-1(ok1387)* mutants and WT animals (Fig. 6 and 7), suggesting that NMUR-1′s metabolic regulation is specifically engaged during infection rather than being constitutively active. This supports a model where pathogen detection (via CAPA-1 or bacterial ligands) triggers NMUR-1 signaling and the receptor’s control of ATP synthase is context-dependent, preventing detrimental metabolic dysregulation under homeostatic conditions. Due to its roles in host-pathogen interactions and the highly conserved nature of NMUR-1 across multiple species, future work studying the mechanisms and signaling pathways of NMUR-1 during pathogen infection would provide valuable insights into the communication between the nervous system and other nonneuronal tissues under pathogenic and non-pathogenic conditions**.**

## MATERIALS AND METHODS

### Nematode strains

The following *C. elegans* strains were maintained as hermaphrodites at 20°C, grown on modified Nematode Growth Media (NGM) (0.35% instead of 0.25% peptone) with 15 µg/mL tetracycline, and fed *E. coli* HT115 (60). The wild-type animal strain was *C. elegans* Bristol *N2*. The *atp-3(gk5653)* and *atp-5(gk5424)* were obtained from the *Caenorhabditis* Genetics Center (University of Minnesota, Minneapolis, MN). The *nmur-1(ok1387)* animals were from a previous study performed by our lab. All mutant animals were backcrossed with wild-type Bristol *N2* animals at least three times before experimental use. All genotypes were confirmed using PCR or visual inspection of GFP expression in the pharyngeal bulb for *atp-3(gk5653)* and *atp-5(gk5424)*. All animals used for this study were synchronized 65-h-old young adults, unless otherwise noted in the specific methodology.

### Bacterial strains

The following bacterial strains were grown using standard conditions (61). *E. coli* strain HT115, *S. enterica* strain SL1344, *E. faecalis* strain OG1RF, *E. coli* HT115, and *S. enterica* SL1344 were cultured in Luria-Bertani (LB) broth and agar, which was prepared following the manufacturer’s recommendations. All assays for *E. coli* HT115 and *S. enterica* were performed on NGM. *E. faecalis* OG1RF was cultured in brain heart infusion (BHI) broth and agar, which was prepared following the manufacturer’s recommendations. All assays using *E. faecalis* OG1RF were performed on BHI media containing 10 µg/mL of gentamycin.

### *C. elegans* pathogen exposure and protein collection

Gravid adult wild-type and *nmur-1(ok1387)* animals were lysed using a solution of sodium hydroxide and bleach (volume ratio 5:2), washed using M9 buffer, and eggs were synchronized for 22 h in S-basal liquid medium at room temperature. Synchronized L1 larval animals were transferred onto modified NGM plates seeded with *E. coli* HT115 and grown at 20°C for 48 h until the animals had reached the L4 larval stage. The synchronized wild-type and *nmur-1(ok1387)* L4 larval stage animals were collected and transferred to plates seeded with either *S. enterica* SL1344, *E. faecalis* OG1RF, or *E. coli* HT115 for 24 h at 25°C. Infected and uninfected controls (exposed to *E. coli* HT115) were collected and washed five times in M9 buffer containing a 1× concentration of protease inhibitors (Halt-protease inhibitor cocktail, ThermoFisher Sci.) to remove any bound bacteria. After the last wash and removal of the supernatant, each sample was snap frozen in a bath of ethanol and dry ice. Five biological replicates of *S. enterica*, *E. faecalis*, and *E. coli*-treated animals were collected, respectively. The frozen samples were submitted to the Tissue Imaging and Proteomics Laboratory at Washington State University (Pullman, WA) for mass spectrometry-based quantitative proteomics analyses.

### Protein sample preparation

The worm pellet was freeze-dried and ground into fine powder using a single 2.8  mm i.d. steel ball with a TissueLyser II (Qiagen, Valencia, CA) at a frequency of 30  Hz for 30 s, followed by the addition of 100 µL extraction solvent of PBS buffer, pH 7.5, with protease inhibitor and vortexing. Supernatants were collected after centrifugation at 16,000  ×  *g* (10 min, 4°C). The protein was then quantified with a Qubit Protein Assay Kit (Life Technologies, Carlsbad, CA) in compliance with the manufacturer’s protocol. Disulfide bonds were reduced using 100 mM dithiothreitol (DTT) at a ratio of 1:10 DTT/sample volume and incubated at 50 °C for 45 min. Cysteine bonds were then alkylated with 200 mM iodoacetamide at the same volume ratio for 20 min at room temperature. Finally, the protein was digested with trypsin (G-Biosciences, St. Louis, MO) at a 1:50 ratio of trypsin/protein and incubated at 37°C for 12 h.

### High-resolution nano-HPLC tandem mass spectrometry analysis

The peptide samples were subjected to Thermo Scientific Orbitrap Fusion Tribrid with an Easy-nLC 1000 ultra-high pressure LC on a Thermo Scientific PepMap 100 C18 column (2 µm, 50 µm  ×  15 cm). The peptides were separated over a 115 min gradient eluted at 400 nL/min with 0.1% formic acid (FA) in water (solvent A) and 0.1% FA in acetonitrile (solvent B) (5%–30% B in 85 min, followed by 30%–50% B over 10 min and 50%–97% B over 10  min). The run was completed by holding a 97% B for 10 min. MS1 data were acquired on an Orbitrap Fusion mass spectrometer using a full scan method according to the following parameters: scan range 400–1,500 m/z, Orbitrap resolution 120,000, AGC target 400,000, and maximum injection time of 50 ms. MS2 data were collected using the following parameters: rapid scan rate, HCD collision energy 35%, 1.6 m/z isolation window, AGC 2,000, and a maximum injection time of 50 ms. MS2 precursors were selected for a 3 s cycle. The precursors with an assigned monoisotopic m/z and a charge state of 2–7 were interrogated. The precursors were filtered using a 60 s dynamic exclusion window. MS/MS spectra were searched using Thermo Scientific Proteome Discoverer software version 2.0 with SEQUEST against the Uniprot *Caenorhabditis elegans* database (TaxID  =  6239). Precursor and fragment mass tolerances were set to 10 ppm and 0.8 Da, respectively, and allowed up to two missed cleavages. The static modification used was carbamidomethylation (C). The high confidence level filter with a false discovery rate (FDR) of 1% was applied to the peptides. Protein relative quantitation was achieved by extracting peptide areas with the Proteome Discoverer 2.0 (Thermo Scientific, San Jose, CA), and three unique peptides per protein were used for the protein quantitation analysis. The mass spectrometry proteomics data have been deposited to the ProteomeXchange Consortium via the PRIDE65 partner repository with the data set identifier PXD045826.

### Ontology analysis

The results of the untargeted proteomics screen were first sorted by FDR to remove any results that had an FDR score lower than 1% to obtain the total proteins identified with high confidence. Next, proteins were sorted by their abundance ratio comparing the proteomes of either infected versus uninfected animals or *nmur-1(ok1387)* mutant animals versus WT animals. Proteins were considered upregulated if the abundance ratio of a protein was 1.5 or greater, and proteins were considered downregulated if the abundance ratio was 0.75 or less. Potentially regulated proteins were sorted again by their adjusted *P*-values to eliminate any non-significant different abundance ratios to form the final list of significantly regulated proteins. The lists of significantly regulated proteins were analyzed using the online software GOrilla (https://cbl-gorilla.cs.technion.ac.il/) along with the total proteins discovered as background using the mode “Two unranked lists of genes” (62). The GO analyses were also performed using the *C. elegans*-specific enrichment tools available on WormBase (https://wormbase.org//tools/enrichment/tea/tea.cgi) (63, 64) to validate the results obtained with GOrilla.

### Survival assay

Wild-type and mutant animals were synchronized by egg-laying. Briefly, well-fed gravid adult animals were transferred to fresh *E. coli* HT115-seeded NGM plates and incubated for 45 min at 25°C. Adult animals were removed after 45 min, and the synchronized offspring were grown at 20°C for 65 h to reach the young adult stage. Bacterial lawns for the survival assays were prepared by culturing pathogenic bacteria for 15−16 h in either LB broth for *S. enterica* or BHI broth for *E. faecalis* OG1RF at 37°C in a shaking incubator. A 30 µL drop of the fresh bacterial culture was placed on 3.5 cm plates of either modified NGM or BHI media with 10 µg/mL of gentamicin. Plates were incubated at 37°C for 15−16 h, cooled to room temperature, and then seeded with synchronized 65-h-old young adult animals. The survival assays were performed at 25°C, and live animals were transferred daily to fresh plates until egg laying ceased. Animals were scored at the times indicated and were considered dead when they failed to respond to touch.

### Quantification of intestinal colonization

Synchronized 65-h-old animals were prepared using the method described above. GFP-tagged bacteria were prepared using the method described above with the addition of antibiotics in the media (*S. enterica* SL1344::*gfp* with 50 µg/mL of kanamycin, *E. faecalis* OG1RF::gfp with 100 µg/mL of rifampicin). Twenty-four hours prior to pathogen exposure, the synchronized animals were transferred to NGM with DMSO or 20 µM of DCC in the media. After this period, animals were transferred to plates seeded with GFP-tagged bacteria with either DMSO or 20 µM of DCC in the media. The animals were incubated at 25°C for 24 h. After 24 h, 10 animals were transferred to an empty NGM plate and allowed to crawl to remove excess external bacteria for 10 min. After 10 min, the animals were picked using *E. coli* OP50 and transferred to a 1.5 mL tube filled with 100 µL of PBS + 0.1% TritonX, 30 mM sodium azide, 1 mg/mL ampicillin, and 1 mg/mL of tetracycline. The animals were allowed to soak in the antibiotic solution for 45 min to eliminate any residual external bacteria. After soaking, the animals were washed three times using PBS + 0.1% Triton X to remove the antibiotics. The animals were then ground for 30 s using a motorized pestle to free the bacteria colonizing the intestine. Serial dilutions of the resulting lysate (10^−1^, 10^−2^, 10^−3^, and 10^−4^) were plated on antibiotic selective media and incubated for 24 h at 37°C.

### Bacterial growth curve quantification

The growth curves of *S. enterica* SL1344 and *E. faecalis* OG1RF were measured following a previously published protocol (65). In brief, individual colonies from an isolation streak were used to inoculate 5 mL of liquid media, which were then grown at 37°C for 16 h. The fresh liquid culture of bacteria was diluted 100× by combining 60 µL of the bacterial culture with 6 mL of sterile liquid media. The diluted bacterial culture was split into two groups with either DMSO or 20 µM of DCC; 100 µL of each of the bacterial cultures was pipetted into three wells of a 96-well plate, and the plate was loaded into a microplate reader set to 37°C. Optical density was measured for at least 21 h, with measurements being taken every 10 min at a wavelength of 595 nm. *S. enterica* was grown in LB broth without the presence of antibiotics in either the DMSO or DCC condition. *E. faecalis* was grown in BHI broth with 15 µg/mL of gentamicin in both the DMSO and DCC conditions.

### RNA interference

RNAi was conducted using the Ahringer group library and feeding synchronized L4 larval *C. elegans E. coli* strain HT115(DE3) expressing double-stranded RNA (dsRNA) that was homologous to a target gene (66, 67). Before exposure, all RNAi clone plasmids were isolated, digested with KpnI, and Sanger sequenced using a T7 promoter primer to check for gene specificity. *E. coli* with the appropriate dsRNA vector was grown in LB broth containing ampicillin (100 µg/mL) at 37°C for 15−16 h, and 120 µL was plated on modified NGM plates containing 100 µg/mL ampicillin and 3 mM isopropyl β-D-thiogalactoside (IPTG). The bacteria were allowed to grow for 15−16 h at 37°C. The plates were cooled away from direct light before the synchronized L3 larval animals were placed on the bacteria. Synchronized L4 larvae worms were placed on RNAi or vector control plates for 24 h at 20°C. Adults were then transferred to survival assay plates. Clone identity was confirmed by sequencing at Eton Bioscience Inc. *unc-22* RNAi was included as a positive control in all experiments to account for RNAi efficiency.

### ATP quantification

Synchronized wild-type and *nmur-1(ok1387)* animals and bacteria plates were prepared as described in the survival assay; 65-h-old animals were placed on either *S. enterica* SL1344 or *E. faecalis* OG1RF plates. The animals were incubated at 25°C for 24 h. Following incubation, animals were randomly picked from the bacterial plates, placed into 50 µL of M9 buffer, and washed three times, then snap frozen in a bath of dry ice and ethanol. Suspended animals were boiled for 15 min at 95°C, then placed on ice for 5 min. Supernatant was collected and transferred to a new 1.5 mL tube after centrifugation at 14,800 × *g* for 10 min at 4°C. ATP quantification was performed using the ATP Determination kit by ThermoFisher (A22066) following the manufacturer’s protocol using 10 µL of 10-fold diluted supernatant in a 100 µL reaction. Luminescence was recorded using a Molecular Devices FilterMax F5.

### *N,N'*-Dicyclohexylcarbodiimide treatment

NGM and BHI media were prepared following the method described above with the addition of either DMSO or 20 µM of *N,N'*-Dicyclohexylcarbodiimide (DCC). A 20 mM solution of DCC was prepared by dissolving 41.266 mg of DCC into 10 mL of DMSO. The solution was prepared fresh and not allowed to be stored for more than 1 day, as the combination of DCC and DMSO causes the DCC to undergo Pfitzer-Moffatt oxidation and precipitate out of solution; 1 mL of DMSO or 1 mL of 20 mM DCC in DMSO was added to 1 L of media before it solidified. Animals were synchronized as described above in the absence of DCC. Synchronized animals were moved to DCC-containing NGM media plates 24 h before pathogen exposure. The survival assays were carried out with the designated pathogen on plates that also contained DCC in the same manner as described above.

### Quantification of H_2_O_2_ production

H_2_O_2_ levels in worms were quantified following a previously described protocol (68). In short, synchronized wild-type and *nmur-1(ok1387)* animals and bacteria plates were prepared using the bleach syncing method as described above; 65-h-old animals were collected in M9 buffer and placed on *E. faecalis* OG1RF plates, then placed in a 25°C incubator for 24 h. After 24 h, the animals were washed off the bacteria plates using M9 buffer, and the adult animals were allowed to settle to the bottom of a 15 mL conical tube before the supernatant was removed. The animals were washed twice to remove excess bacteria and L1 larval animals. The animal pellet was then washed twice with 5 mL of Amplex assay reaction buffer to further remove excess bacteria before being transferred to an empty BHI plate. Thirty washed adult animals were transferred from the BHI plate to a single well of a black, clear-bottom 96-well plate filled with 50 µL of 1× Amplex assay reaction buffer. Fifty microliters of Amplex working solution was then added to each well. The final concentration of reagents was 50 µM of Amplex Red and 0.1 U/mL of horseradish peroxidase. Animals were placed on the opposite end of the assay plate away from the standards to avoid cross-contamination. The assay was measured over the course of 2 h using a Molecular Devices FilterMax F5; measurements were taken every 15 min.

### ROS quantification using 2’,7’-dichlorodihydrofluorescein diacetate

ROS quantification using 2’,7′-dichlorodihydrofluorescein diacetate (H_2_DCFDA) was performed following a previously described protocol (47). In short, synchronized wild-type and *nmur-1(ok1387)* animals and bacterial plates were prepared using the egg-laying method as described above. Sixty-five-hour-old animals were collected in M9 buffer and were placed on either *S. enterica* SL1344 or *E. faecalis* OG1RF plates. The plates were then placed in a 25°C incubator for 24 h. After 24 h, the animals were transferred to an unseeded NGM plate and allowed to crawl to separate the adults from both eggs and L1 larval animals. Thirty adult animals were transferred to a single well of a black, clear-bottom 96-well plate filled with 50 µL of M9 buffer. Fifty microliters of 50 µM H_2_DCFDA was pipetted into each well for a final concentration of 25 µM. The assay was measured over the course of 6 h using a Molecular Devices FilterMax F5, and measurements were taken every 30 min. This extended monitoring ensured that H₂DCFDA fully reacted to any ROS present in the well. A representative time point between 3 and 4 h was selected for analysis, as this was the earliest interval at which reproducible differences between strains were observed.

### Western blots

Western blots were performed as previously described (69). In brief, equal masses of wild-type and *nmur-1(ok1387)* whole lysate were diluted with H_2_O to 7.5 µL, mixed with 2.5 µL of NuPAGE LDS buffer, and heated to 70°C for 10 min for denaturation. The lysates were separated on a NuPAGE 4%–12% Bis-tris gel and transferred to a nitrocellulose membrane. The membrane was blocked using 30 mL of 5% skim milk in Tris-buffered saline + 0.1% Tween 20 (TBST) for 1 h at 20°C. Following blocking and between each subsequent step, the membrane was washed three times with 20 mL of TBST for 5 min. Primary antibodies used were mouse anti-HSP-60 (DSHB, HSP60) diluted in 1× PBS to 1:10, rabbit anti-ATP-4 sera (produced for this study by ThermoFisher) diluted in 5% skim milk to 1:2,000, and mouse anti-tubulin antibody (Sigma Aldrich, T6074) diluted in 5% skim milk to 1:10,000 for 16 h at 4°C under gentle agitation. The secondary antibodies used were goat anti-mouse IgG (H + L) antibody conjugated to HRP (Promega, W4021) and goat anti-rabbit IgG (H + L) antibody conjugated to HRP (Promega, W4011). Secondary antibodies were diluted 1:2,500 for the detection of anti-HSP60 and anti-ATP-4; 1:10,000 for the detection of anti-tubulin in 5% skim milk in TBST. Membranes were incubated under gentle agitation for 1 h at 20°C. Band detection was done using a Pierce ECL kit (Thermofisher, catalog 32106), and the immunoblots were imaged using the iBright 1500 system (Thermofisher, catalog #A44241). Antibody specificity for HSP-60 and ATP-4 was validated by RNAi-mediated knockdown, as homozygous knockout mutants for these genes are lethal or developmentally arrested.

### Quantification and statistical analysis

Survival curves were plotted using GraphPad PRISM (version 10) computer software. Survival was considered different from the appropriate control indicated in the main text when *P* < 0.05. PRISM uses the product limit or Kaplan-Meier method to calculate survival fractions and the log-rank test, which is equivalent to the Mantel-Haenszel test, to compare survival curves, and qRT-PCR results were analyzed using two-sample *t*-tests for independent samples; *P* values < 0.05 are considered significant. All experiments were repeated at least three times, unless otherwise indicated. Statistical details for each figure are listed in its corresponding figure legend.

## Data Availability

The *C. elegans* strains and recombinant DNA generated in this study will be shared upon request, but we may require payment to cover shipment and completion of a Material Transfer Agreement for possible commercial applications. The raw proteomic data were deposited to the Proteome Exchange via Proteomics IDEntifications database (PRIDE) with the accession number PXD045826. No original code was created during the course of this study. Any additional information required to reanalyze the data reported in this paper is available from the lead contact upon request.

## SUPPLEMENTAL MATERIAL

Supplemental material iai.00313-25-s0001.pdf Fig. S1 to S7.

Table S1 iai.00313-25-s0002.xlsx Overlapping genes and proteins in transcriptomic and proteomic data.

ASM does not own the copyrights to Supplemental Material that may be linked to, or accessed through, an article. The authors have granted ASM a non-exclusive, world-wide license to publish the Supplemental Material files. Please contact the corresponding author directly for reuse.
