# Supplementary material for: The GPCR NMUR-1 mediates neural regulation of energy homeostasis in response to pathogen infection
Source: Infect Immun. 2025 Nov 3;93(12):e00313-25. doi: 10.1128/iai.00313-25 (PMC12707106; doi:10.1128/iai.00313-25)
Supplement: Supplemental material — Fig. S1 to S7. [file iai.00313-25-s0001.pdf]

Supplementary Figures

Figure S1

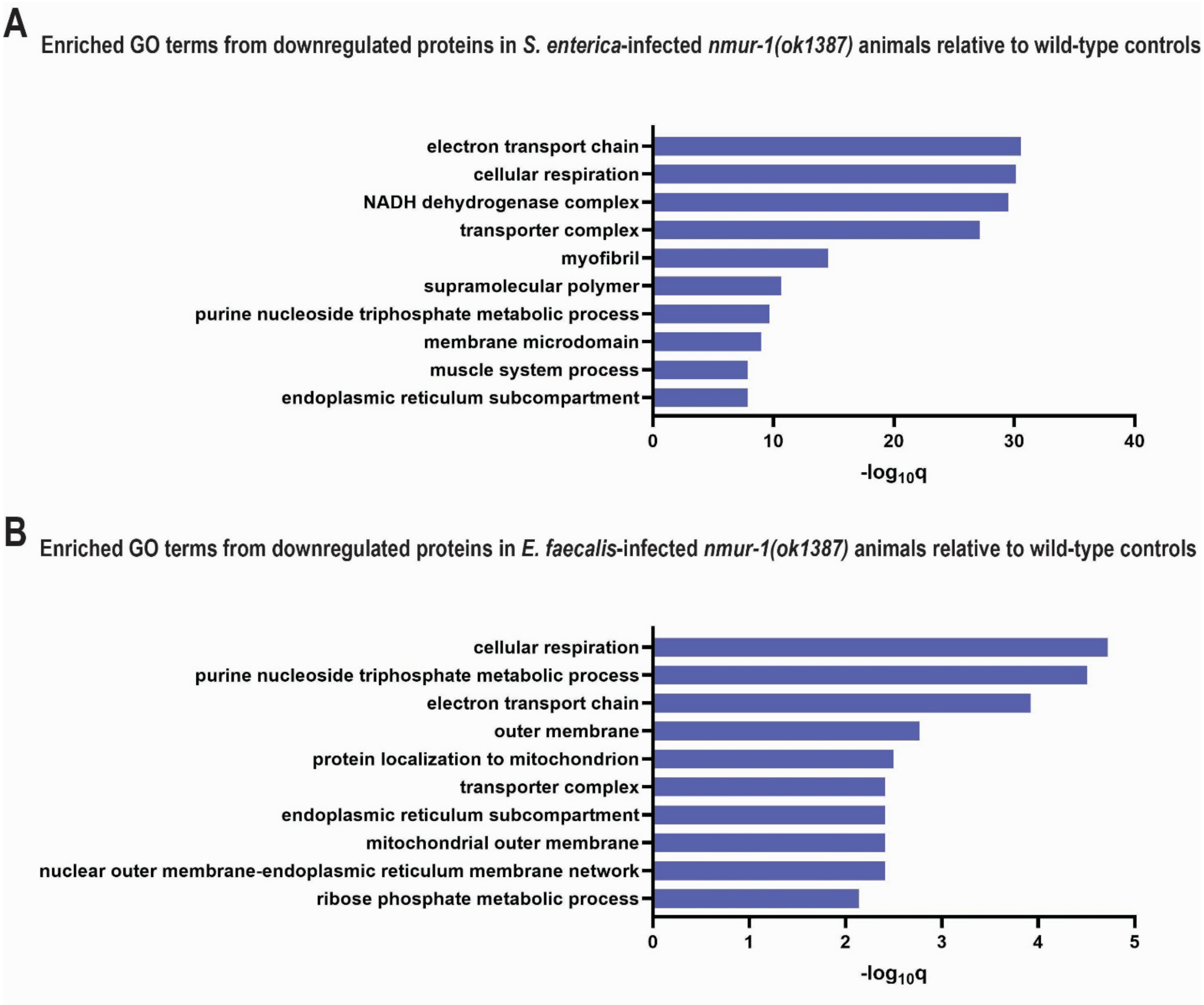

Figure S1. *nmur-1(ok1387)* mutant animals had significant downregulation in electron transport and energy production proteins during infection. Downregulated proteins in *nmur-1(ok1387)* animals infected with *S. enterica* **(A)** or *E. faecalis* **(B)** relative to infected WT control animals were subjected to GO analysis using the *C. elegans*-specific enrichment tools available on WormBase (https://wormbase.org//tools/enrichment/tea/tea.cgi). The graphs show the top 10 most significantly enriched GO terms in mutant animals. Bars represent the significance levels of enrichment expressed as $-\log_{10}q$ values.

Figure S2

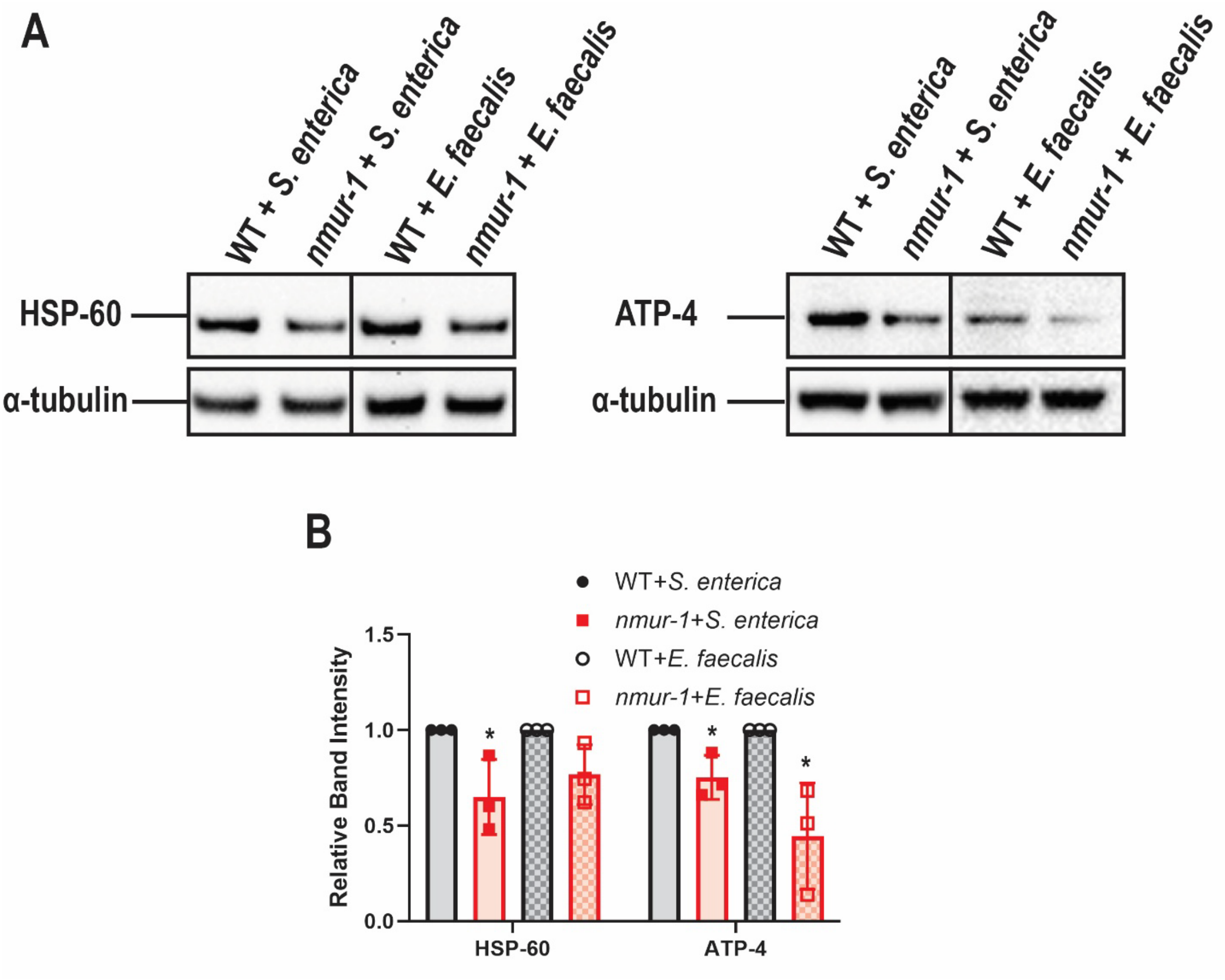

Figure S2. NMUR-1 regulates protein expression of HSP-60 and ATP-4 during *S. enterica* and *E. faecalis* infections. (A) Western blot of the whole worm lysates prepared from WT and *nmur-1(ok1387)* animals exposed to either *S. enterica* or *E. faecalis* was probed for the expression of HSP-60 or ATP-4. Alpha-tubulin was probed as a loading control. **(B)** Bar graph represents the alpha-tubulin normalized expression of HSP-60 and ATP-4 in *nmur-1(ok1387)* animals relative to WT animals. The graph is the combination of three independent replicates. Asterisk (*) denotes a significant difference ($p < 0.05$) between WT and *nmur-1(ok1387)* animals, as analyzed using Student's *t*-test.

Figure S3

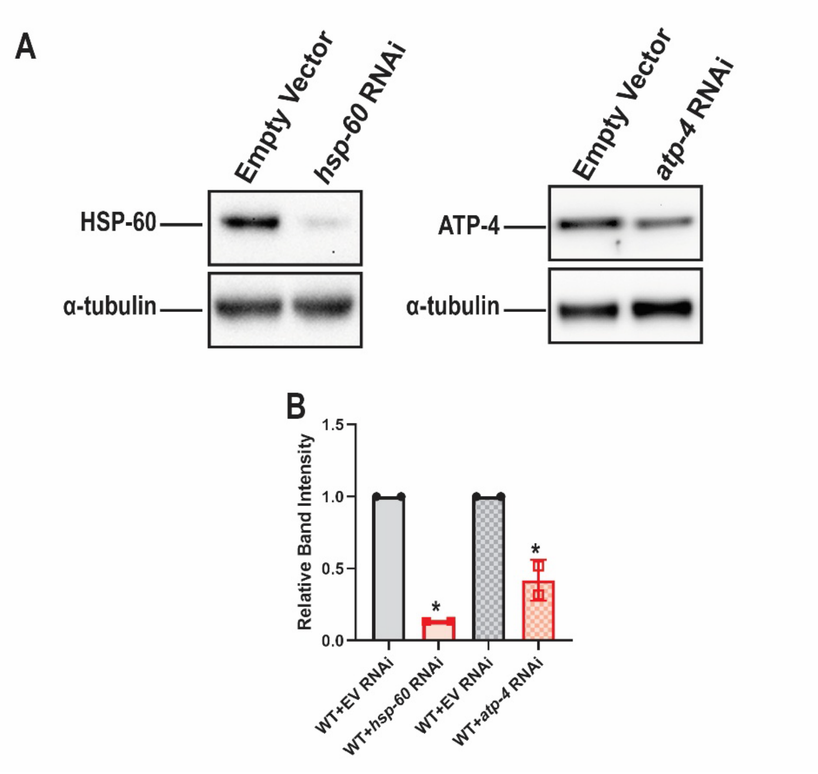

Figure S3. Validation of antibodies against HSP-60 and ATP-4. Western blot of the whole worm lysates prepared from WT animals grown on dsRNA for *hsp-60*, *atp-4*, or empty vector (EV) control was probed for the expression of HSP-60 or ATP-4. α-tubulin was probed as a loading control. **(B)** Bar graph represents the a-tubulin normalized expression of HSP-60 and ATP-4 in WT animals with *hsp-60* or *atp-4* RNAi relative to EV control. The graph is the combination of two independent replicates. Asterisk (*) denotes a significant difference ($p <$ 0.05) between *hsp-60* or *atp-4* RNAi and EV control, as analyzed using Student's *t*-test.

Figure S4

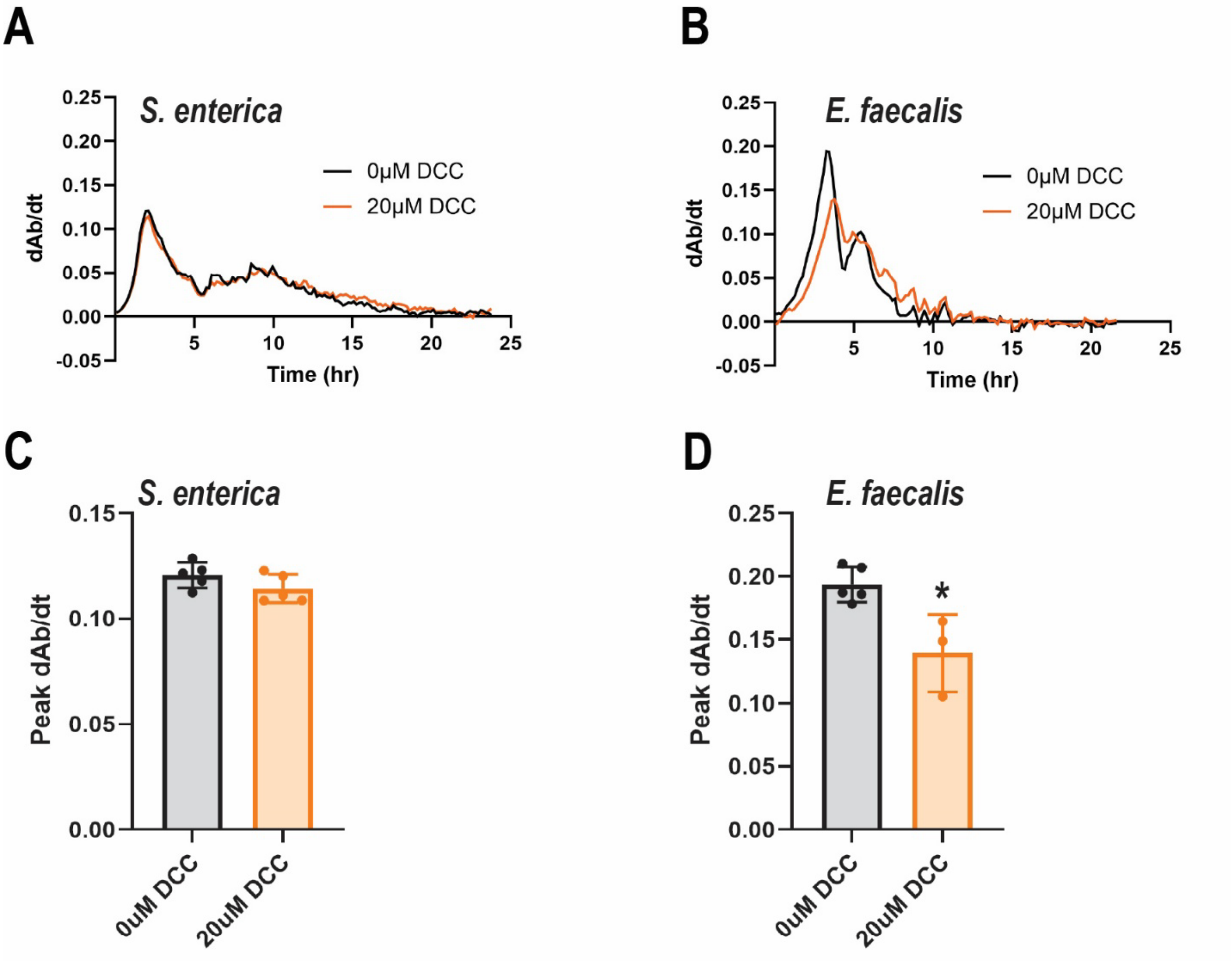

Figure S4. *N,N′*-Dicyclohexylcarbodiimide (DCC) inhibited the growth of *E. faecalis* but had no significant effects on the growth of *S. enterica*. *S. enterica* **(A)** or *E. faecalis* **(B)** growth curves were measured over time in the presence of either DMSO or 20µM of DCC. The graphs are the 1st order derivative of the combination of three independent experiments. The quantifications of the maximal rates of growth for *S. enterica* and *E. faecalis* were shown in **(C)** and **(D)**, respectively. Asterisk (*) denotes a significant difference ($p < 0.05$) between DMSO and DCC treatment, as analyzed using Student's *t*-test.

Figure S5

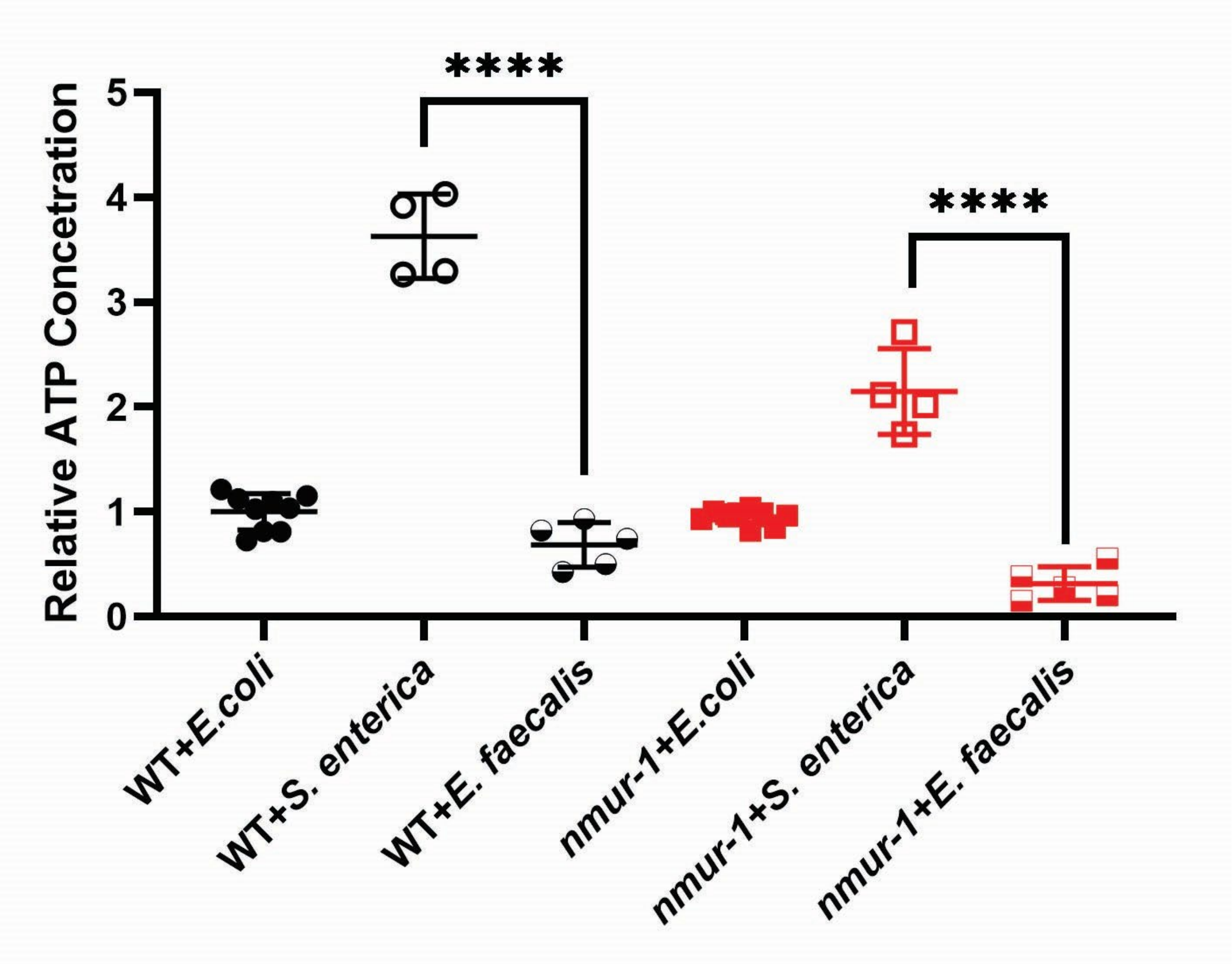

Figure S5. *S. enterica* infection increased ATP concentrations compared to *E. faecalis* infection. WT and *nmur-1(ok1387)* animals were exposed to *E. coli*, *S. enterica*, or *E. faecalis* for 24 hours followed by ATP concentration measurement. All ATP concentrations were normalized to the uninfected WT *E. coli* control. The graphs show combined results of at least 4 independent experiments. In each experiment, 30 animals of each strain under either condition were used. Error bars represent standard deviation. Asterisks (****) denote a significant difference ($p < 0.0001$) between *S. enterica* and *E. faecalis* infections, as analyzed using Dunnett's multiple comparison test.

Figure S6

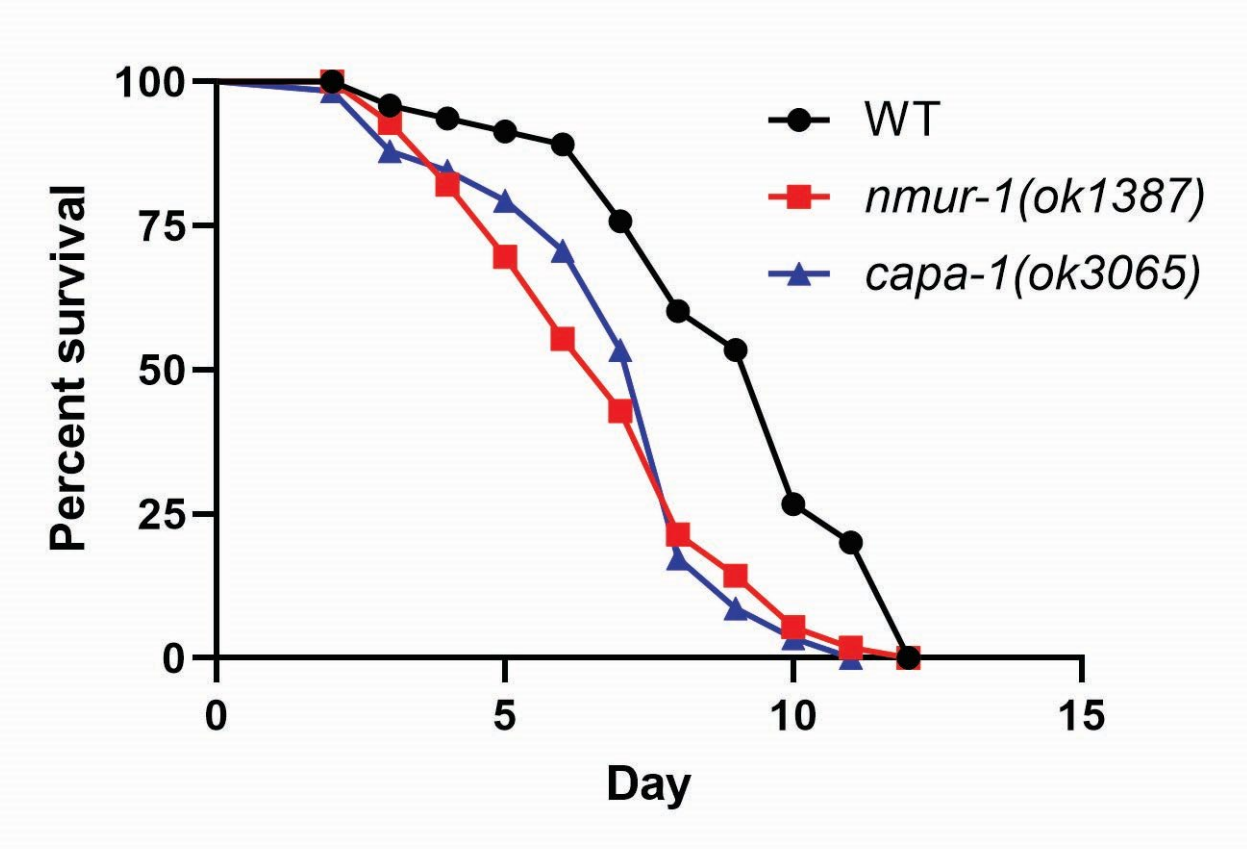

Figure S6. Loss of CAPA-1 reduced *C. elegans* survival against *E. faecalis*, similar to the survival phenotype of *nmur-1(ok1378)* animals. WT, *nmur-1(ok1387)*, and *capa-1(ok3065)* animals were exposed to *E. faecalis* and scored for survival over time. The graphs show a representative of three independent replicates. Each experiment included $n = 60$ animals per strain. p values represent the significance levels of mutants relative to WT: *nmur-1(ok1387)*, $p < 0.0001$; *capa-1(ok3065)*, $p < 0.0001$.

Figure S7

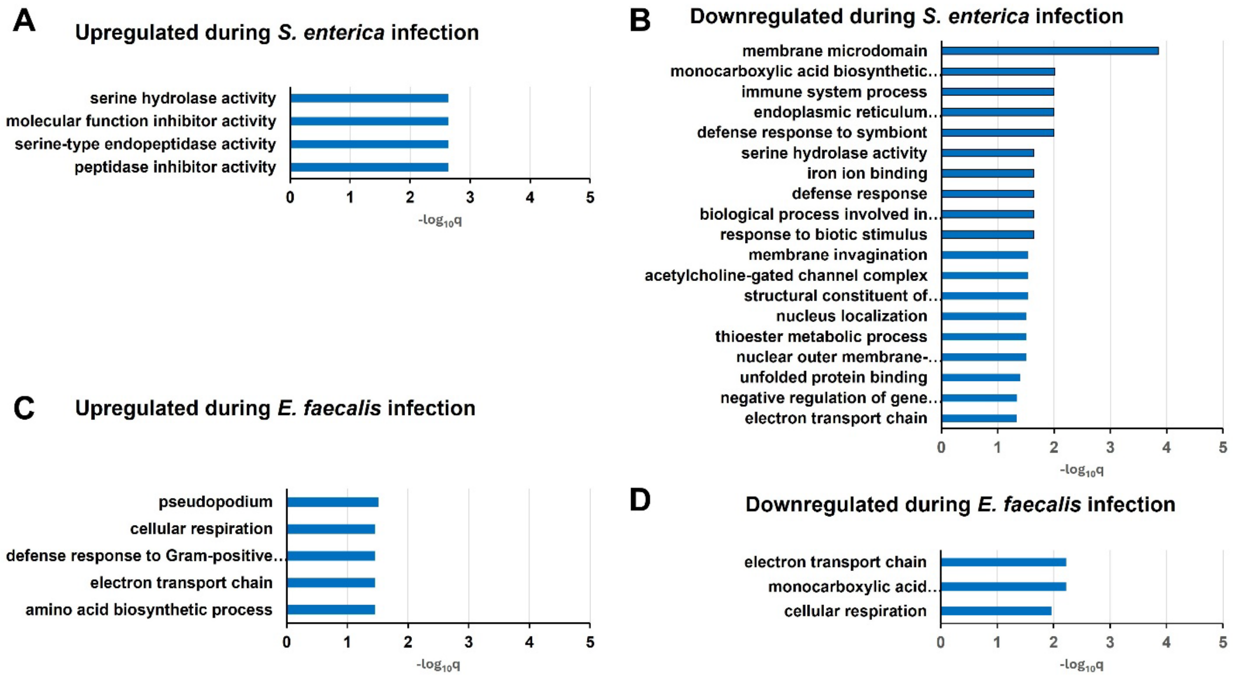

Figure S7. GO analysis on the overlapping genes and proteins in our transcriptomic and proteomic data. Overlapping genes and proteins in each category were listed in Table S1. GO analysis was performed using the *C. elegans*-specific enrichment tools available on WormBase (https://wormbase.org//tools/enrichment/tea/tea.cgi). Bars represent the significance levels of enrichment expressed as $-\log_{10}q$ values.
